# Prevalence of adverse events in hip and knee arthroplasties following the implementation of surgical checklists*

**DOI:** 10.1590/1518-8345.7658.4557

**Published:** 2025-05-19

**Authors:** Josemar Batista, Elaine Drehmer de Almeida Cruz

**Affiliations:** 1 Universidade Federal do Paraná, Departamento de Enfermagem, Curitiba, PR, Brazil; 2 Universidade Positivo, Escola de Ciências da Saúde, Curitiba, PR, Brazil

**Keywords:** Perioperative Care, Medical Errors, Patient Safety, Arthroplasty, Checklist, Outcome and Process Assessment Health Care

## Abstract

to identify the prevalence of adverse events in patients undergoing hip and knee arthroplasties following the implementation of surgical checklists.

an evaluative study, based on effect analysis, conducted over three periods: pre- (0- 2010) and post- intervention (I- 2013; II- 2016), with retrospective consultation of a simple random sample of 291 medical records between November 2020 and March 2022. The Canadian Adverse Events Study and Global Trigger Tool forms were used to track and confirm adverse events. Cases were analyzed using descriptive and inferential statistics; p-values ≤ 0.05 indicated significance.

in the post-implementation periods of surgical checklists, a reduction was observed in the frequency of patients affected by two or more events, from 27.8% to 11.3% (p = 0.002), and in the overall prevalence, from 63.9% to 36.1% (p < 0.001). A decrease in the prevalence of patients affected by urinary retention (33% to 3.1%; p < 0.001) and hemorrhage (9.3% to 0%; p = 0.012) was also noted. There was an increase in the prevalence of skin lesions, from 2.1% to 10.3% (p = 0.043).

there was a reduction in the overall prevalence and frequency of adverse events in patients undergoing arthroplasty following the implementation of surgical checklists.

## Highlights

**(1)** High prevalence of adverse events in hip and knee arthroplasties. **(2)** Surgical checklists reduce the prevalence of adverse events in arthroplasties. **(3)** There was a significant decrease in patients affected by two or more adverse events. **(4)** Investigating the impact of interventions contributes to fostering a culture of safety. **(5)** The results are relevant for strengthening basic perioperative safety measures.

## Introduction

Evidence that surgical mortality is higher among those receiving lower-quality care compared to those without access to surgical services reiterates the importance of innovative actions aimed at providing better practices in the perioperative context^(1-2)^. This finding underscores the critical nature of the sector, both in terms of task- and process-related demands and the cognitive demands on healthcare professionals, to ensure the delivery of high-quality care^(2)^.

It is recognized that the complexity of the surgical field contributes to the occurrence of errors and incidents related to care, especially in interventions that require the use of tools and implants, such as knee and hip arthroplasties^(3-5)^. Conceptually, an error consists of failing to execute a planned action as intended or incorrectly implementing a plan. These occurrences increase the risk of incidents that cause harm to the patient, defined as adverse events (AE)^(6-7)^.

In orthopedic care, regardless of the type of anesthesia and surgical procedure the patient undergoes, errors are not uncommon. There is consensus that this surgical specialty has a higher probability of preventable harm compared to others^(3,8-9)^. There is a greater likelihood of wrong-site surgeries^(10)^ and severe infectious events requiring reoperation^(11-12)^, mainly due to the particularities inherent to the implantation of materials^(13)^. The lack of standardization of equipment, procedures and protocols in this specialty adds to the risks and increases the occurrence of harm^(14)^.

Given the various critical factors that contribute to surgical errors and AE, the World Health Organization launched the Safe Surgery Saves Lives (SSSL) program in the 2007-2008 biennium, recommending the use of surgical checklists and their optional adaptation to meet local and regional demands. The instrument, in its original version, contains 19 items and aims to ensure patient safety^(15-16)^. Since then, research has highlighted the benefits of this tool, leading to a 33% reduction in AE^(16-17)^, with results that reinforce its importance in promoting care quality and preventing errors.

In this context, safety actions aimed at meeting the objectives of the aforementioned program were gradually adopted at the hospital of this study. In 2011, the surgical checklist recommended by the WHO was adapted and pioneeringly used by the orthopedic specialty in surgeries involving implants^(18)^. In the second half of 2014, another checklist was made available for use in the orthopedic inpatient unit, aimed at verifying safety items related to the pre- and postoperative phases^(19)^.

When analyzing the human and financial resources committed to the surgical area, as well as the adaptations and/or development of checklists for the consolidation of institutional actions and policies in the hospital under study, it was considered important to conduct evaluative studies. These studies aim to assess the effects of implementing these tools in the healthcare context, through pre- and post-intervention analysis.

Therefore, the objective of this research was to identify the prevalence of adverse events in patients undergoing hip and knee arthroplasties following the implementation of surgical checklists.

## Method

### Study design

This is an evaluative study of the effects analysis type^(20)^, documentary, retrospective, and with a quantitative approach.

### Study setting

The research was conducted at a large teaching hospital in the southern region of Brazil, which provides care exclusively through the *Sistema Único de Saúde*. The surgical specialty involved was orthopedics, and the data from hospitalizations of patients undergoing hip and knee arthroplasties in distinct periods, corresponding to before and after the implementation of two surgical checklist modalities, were considered. These periods are referred to here as pre-intervention and post-interventions I and II. The choice of this population, specifically, is justified by the surgical complexity of the procedure involving prosthesis implantation and the pioneering role of the specialty in using these instruments.

The Intervention I occurred in 2011 with the implementation of a checklist for use in the surgical center, composed of 45 items organized into four phases: (I) patient reception, (II) before anesthetic induction, (III) before surgical incision, and (IV) before the patient leaves the operating room^(18)^. In 2014, a checklist was implemented for use in the orthopedic inpatient unit (Intervention II), containing 97 safety items distributed across six phases: (1) Identification; (2) Preoperative: items to be verified before the patient is sent to the surgical center; (3) Immediate Postoperative: verification of safety indicators within the first 24 hours after the anesthetic-surgical procedure; (4) Intermediate Postoperative: items related to pain assessment, evaluation of the surgical wound, and restoration of physiological systems; (5) Complications: recording of postoperative complications, such as infections; and (6) Hospital discharge/transfer: safety items related to the patient’s general condition, surgical site, and devices, as well as guidance for home care and outpatient follow-up^(19)^.

Both checklists include the verification of elements that contribute to error prevention and the detection of non-conformities related to safety and care quality. A two-year interval was adopted for investigation and analysis, corresponding to the year 2010 (pre-intervention) and the years 2013 and 2016 (post-intervention I and II).

### Population and selection criteria

The source of information for the study was the database provided by the hospital’s information technology department, consisting of all hip and knee arthroplasty surgeries performed from January 1 to December 31, 2010 (pre-intervention), January 1 to December 31, 2013 (post-intervention I), and January 1 to December 31, 2016 (post-intervention II). Duplicate medical records were eliminated, keeping only those related to the first surgery performed during the analyzed hospitalization, corresponding to the index hospitalization.

The medical records of adult patients with a minimum hospitalization time of 24 hours were included. Cases of deaths occurring in the intraoperative period and with a hospitalization period of less than 24 hours were also included. Cases with a diagnosis related to psychiatric disorders were excluded, as previously established^(21-22)^.

### Sample definition

To estimate the sample size, a pilot study was conducted to detect significant differences in the prevalence of AE when comparing the pre-intervention and post-intervention II periods. The initial prevalence estimate was based on a pilot sample of 25 medical records, randomly selected for each condition, which showed a prevalence of 60% and 40% of AE, respectively, applying the data collection methodology described below. For the sample size calculation, a significance level of 5% and a test power of 80% were considered, resulting in a minimum sample size of 97 medical records for each condition.

The simple random selection of eligible medical records was performed from a general surgery list generated by the institutional database, using Microsoft Office Excel 2016^®^ software. Medical records unavailable in the physical filing service were replaced by the immediately following records from the list, with no irreplaceable losses. The medical records used in the pilot study were included in the research.

### Data collection and instruments used

Data collection was carried out between November 2020 and March 2022 through a retrospective consultation exclusively of information contained in physical medical records, and was systematized in two phases, according to the methodology adapted from the Canadian Adverse Events Study protocol^(21)^. Phase I (primary review) was conducted by a single nurse and principal researcher, consisting of the search and identification of potential AE (pAE), guided by two tracking forms.

The first form corresponds to the Canadian Adverse Events Study protocol, consisting of 17 explicit criteria for tracking pAE related to surgery, anesthesia, medications, diagnosis, care, and non-medication treatments^(21)^. This protocol was translated and adapted for use in Brazil^(22)^. The second tracking form refers to the surgical module of the Global Trigger Tool, developed by the Institute for Healthcare Improvement in the United States of America. It was applied with the aim of expanding the search for AE indicators, consisting of 11 triggers for identifying pAE that occurred during intraoperative and postoperative periods^(23)^, translated and cross-culturally adapted for the Brazilian context^(24)^.

For the identification of pAE occurring after hospital discharge, the records contained in outpatient consultation forms, which are part of the patients’ medical records, were used. In cases of Surgical Site Infection (SSI), the criteria from the Centers for Disease Control and Prevention were adopted, defining it as an infection occurring within 30 days and/or 90 days after a surgical procedure involving implant insertion^(25)^.

In cases where at least one tracking criterion was identified, the pAE assessment form^(21-22)^ and the semi-structured script for characterizing demographic, clinical, surgical, and anesthetic profiles, developed for the research, were completed.

The records from Phase I (primary review) were analyzed in Phase II (secondary review), which corresponded to the step of confirming or discarding the AE through a consensus by a committee of specialists^(21-22)^. This committee was intentionally formed by the principal researcher, a nurse and a physician, all holding master’s degrees and with over 10 years of experience in quality management and patient safety.

To evaluate the pAE, the forms filled out in the previous phase were used. To confirm or discard the case, the WHO concept of AE was adopted^(6-7)^, and three scales were applied. The first assessed the severity of the AE^(6)^. The second and third scales were used to determine, respectively, whether the harm was caused by the care provided to the patient and the degree of preventability of the event. These scales consist of six scores ranging from (1) “practically no evidence” to (6) “practically certain evidence”. AE with scores ≥ 4 points were considered preventable^(21,26)^.

### Study variables

The recorded quantitative and/or categorical variables were: sex, age (in years), surgery classification (elective; emergency), contamination potential (clean; potentially contaminated; contaminated; infected), type of anesthesia (inhalation/sedation; general; spinal; epidural; block; local), and surgical risk according to the American Society of Anesthesiologists (ASA) classification. Additionally, comorbidities (intrinsic risk factors) recorded in the pre-anesthetic evaluation form and the use of medical-hospital devices (extrinsic risk factors) were considered. Information was also collected to measure the patient’s preoperative length of stay (< 24 hours; ≥ 24 hours) and total hospital stay (in days).

AE were classified as: (1) mild; (2) moderate; (3) severe; and (4) death^(6)^. The avoidability of AE was defined using the following subclassification: (A) Strongly avoidable (score 6); (B) Potentially avoidable (scores 4 and 5); (C) Potentially non-avoidable (scores 2 and 3); and (D) Strongly non-avoidable (score 1)^(27)^.

Confirmed AE were also categorized according to the International Classification for Patient Safety in class 1 (type of incident), and distributed into the following categories: (A) Clinical administration; (B) Clinical process/procedure; (C) Documentation; (D) Healthcare-associated infection (HAI); (E) Medication/intravenous fluids; (F) Blood/blood products; (G) Nutrition; (H) Oxygen/gases/vapors; (I) Medical devices/equipment; (J) Behavior; (K) Patient accidents; (L) Infrastructure/location/facilities; and (M) Resources/organizational management^(6)^.

### Data processing and analysis

The collected data were double-entered into a Microsoft Office Excel 2016^®^ spreadsheet. After checking and correcting inconsistencies, the data were analyzed with statistical support using the International Business Machines Corporation™ - Statistical Package for the Social Sciences (IBM™ - SPSS) version 20.0 for Windows.

The measures used were: AE prevalence among patients: [(number of patients with at least one AE/total number of patients) x 100] and proportion of preventable AE: [(number of preventable AE/total number of AE) x 100].

Quantitative variables were described using univariate descriptive statistics, while categorical variables were presented as absolute and relative frequencies. To compare the pre-intervention, intervention I and intervention II periods for quantitative variables, the one-way analysis of variance (ANOVA) model or the non-parametric Kruskal-Wallis test was used. For multiple comparisons, Dunn’s *post-hoc* test was applied, with p-values corrected using the Bonferroni method. The normality of quantitative variables was assessed using the Kolmogorov-Smirnov test. p-values ≤ 0.05 indicated statistical significance.

### Ethical aspects

The research was approved by the institutional ethics committee under opinion number 3.651.686. A waiver was requested for obtaining the Informed Consent Form (ICF) from patients. The reviewers/specialists invited to participate in Phase II formalized their consent by signing the ICF.

## Results

In the primary review of 291 medical records, the mean age (respectively in the pre-intervention, post-intervention I and post-intervention II phases) was 57.2 years (standard deviation ‒ SD ± 14.4), 56.5 (SD ± 15.5) and 59.5 (SD ± 15.4), with no significant difference between the analyzed periods (p = 0.355). Regarding patients’ intrinsic risk factors, no differences were observed between the pre- and post-intervention periods (p = 0.822). The remaining demographic and clinical characteristics are presented in Table 1.

**Table 1 -:** Demographic and clinical profile of patients undergoing hip and knee arthroplasties before and after the implementation of surgical checklists. Curitiba, PR, Brazil, 2022

**Variable**	**Pre-intervention (n=97)**	**Post-intervention I (n=97)**	**Post-intervention II (n=97)**	**p-value** *
	**n (%)**	**n (%)**	**n (%)**	
**Sex**				
Female	56 (57.7)	59 (60.8)	62 (63.9)	0.677
Male	41 (42.3)	38 (39.2)	35 (36.1)	
**Comorbidity/risk factor**				
Systemic arterial hypertension				
Yes	58 (59.8)	50 (51.6)	56 (57.7)	0.484
No	39 (40.2)	47 (48.5)	41 (42.3)	
Smoking				
Yes	15 (15.5)	25 (25.8)	13 (13.4)	0.057
No	82 (84.5)	72 (74.2)	84 (86.6)	
Diabetes *mellitus*				
Yes	12 (12.4)	14 (14.4)	19 (19.6)	0.359
No	85 (87.6)	83 (85.6)	78 (80.4)	
Pneumopathy ^†^				
Yes	10 (10.3)	6 (6.2)	6 (6.2)	0.455
No	87 (89.7)	91 (93.8)	91 (93.8)	
Thyroid diseases ^‡^				
Yes	9 (9.3)	11 (11.3)	7 (7.2)	0.613
No	88 (90.7)	86 (88.7)	90 (92.8)	
Rheumatoid arthritis				
Yes	6 (6.2)	3 (3.1)	3 (3.1)	0.457
No	91 (93.8)	94 (96.9)	94 (96.9)	
Cardiopathy				
Yes	6 (6.2)	7 (7.2)	7 (7.2)	0.948
No	91 (93.8)	90 (92.8)	90 (92.8)	
Hepatitis				
Yes	5 (5.2)	7 (7.2)	4 (4.1)	0.629
No	92 (94.9)	90 (92.8)	93 (95.9)	
Dyslipidemia				
Yes	4 (4.1)	2 (2.1)	5 (5.2)	0.516
No	93 (95.9)	95 (97.9)	92 (94.9)	
Alcoholism				
Yes	4 (4.1)	4 (4.1)	6 (6.2)	0.741
No	93 (95.9)	93 (95.9)	91 (93.8)	
Hemophilia				
Yes	3 (3.1)	10 (10.3)	8 (8.3)	0.135
No	94 (96.9)	87 (89.7)	89 (91.8)	
Neoplasia				
Yes	2 (2.1)	2 (2.1)	1 (1)	-
No	95 (97.9)	95 (97.9)	96 (99)	
Osteoporosis				
Yes	2 (2.1)	2 (2.1)	0 (0)	-
No	95 (97.9)	95 (97.9)	97 (100)	

*Chi-square Test p< 0.05; ^†^Pneumopathy = Asthma, bronchitis, acute pulmonary edema, pulmonary emphysema, chronic obstructive pulmonary disease; ^‡^Thyroid diseases = Hypothyroidism, hyperthyroidism

The average length of hospitalization was 3.7 days (SD ± 1.8) in 2010 (pre-intervention), 4.9 days (SD ± 3.7) in 2013 (post-intervention I), and 4.6 days (SD ± 4.4) in 2016 (post-intervention II), with a significant difference between the periods (p = 0.008), particularly between 2010 and 2013 (p = 0.006).

It was observed, during hospitalization in all analyzed periods, the presence of extrinsic risk factors, with emphasis on the use of peripheral venous catheter (100% in all periods) and epidural catheter (88.7%; 71.1% and 4.1%, respectively). The latter showed a significant reduction (p < 0.001) in its use before and after interventions I and II, respectively. There was a significant reduction in patients undergoing epidural anesthesia (p < 0.001) and sedation (p = 0.008); the opposite occurred for general anesthesia (p = 0.001), as shown in Table 2, along with other characteristics of interest.

**Table 2 -:** Distribution of the surgical-anesthetic and hospitalization profile of patients undergoing hip and knee arthroplasties before and after the implementation of surgical checklists. Curitiba, PR, Brazil, 2022

**Variable**	**Pre-intervention (n=97)**	**Post-intervention I (n=97)**	**Post-intervention II (n=97)**	p-value*
	**n (%)**	**n (%)**	**n (%)**	
**Preoperative diagnosis**				-
Gonarthrosis	36 (37.1)	37 (38.1)	46 (47.4)	
Coxarthrosis	32 (33)	34 (35.1)	36 (37.1)	
Aseptic loosening of the component	12 (12.4)	8 (8.3)	8 (8.3)	
Secondary coxarthrosis	7 (7.2)	3 (3.1)	2 (2.1)	
Secondary gonarthrosis	4 (4.1)	11 (11.3)	3 (3.1)	
Other ^†^	6 (6.2)	4 (4.1)	2 (2)	
**Preoperative length of stay** 0.343				
< 24 hours	91 (93.8)	88 (90.7)	93 (95.9)	
≥ 24 hours	6 (6.2)	9 (9.3)	4 (4.1)	
**Surgical procedure**				0.220
Total hip arthroplasty	44 (45.4)	43 (44.3)	36 (37.1)	
Total knee arthroplasty	37 (38.1)	45 (46.4)	51 (52.6)	
Revision hip arthroplasty	9 (9.3)	8 (8.3)	6 (6.2)	
Revision knee arthroplasty	7 (7.2)	1 (1)	4 (4.1)	
**Surgery classification**				-
Elective	95 (97.9)	97 (100)	97 (100)	
Emergency	2 (2.1)	0 (0)	0 (0)	
**Potential for contamination**				-
Clean	97 (100)	97 (100)	96 (99)	
Infected	0 (0)	0 (0)	1 (1)	
**ASA** ^‡^ **surgical risk**				0.271
I	22 (22.7)	14 (14.4)	12 (12.4)	
II	61 (62.9)	72 (74.2)	70 (72.2)	
III	14 (14.4)	11 (11.3)	15 (15.5)	
**Type of anesthesia** ^§^				
Epidural	86 (88.7)	65 (67)	7 (7.2)	< 0.001
Spinal	73 (75.3)	74 (76.3)	70 (72.2)	0.790
Sedation	71 (73.2)	61 (62.9)	50 (51.6)	0.008
General	21 (21.7)	23 (23.7)	43 (44.3)	0.001
Block	0 (0)	2 (2.1)	0 (0)	-
Local	0 (0)	1 (1)	4 (4.1)	-

*Chi-square Test, p< 0.05; ^†^Other = Femoral neck fracture, breakage of synthetic material, femoral osteonecrosis, periprosthetic fracture, femur fracture, hemophilic arthropathy, operative infection; ^‡^ASA = American Society of Anesthesiologists; ^§^A single patient may undergo more than one type of anesthesia

The prevalence of AE was 63.9%, 50.5%, and 36.1%, respectively, for the pre-intervention, post-intervention I and post-intervention II periods, with a significant reduction (p < 0.001). The detection of AE occurred predominantly during hospitalization, with a significant difference between the pre- and post-interventions periods (p = 0.046).

**Figure 1 -:**
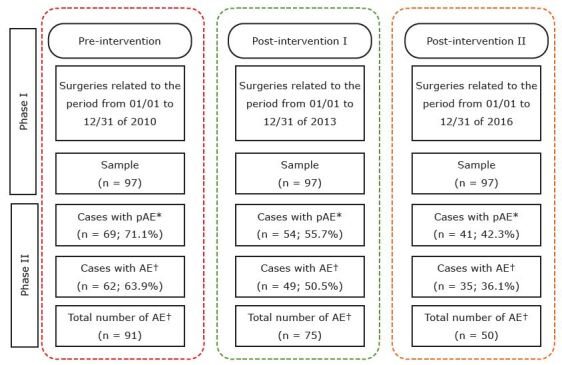
Flowchart of the medical record review and estimation of the prevalence of adverse events before and after the implementation of surgical checklists. Curitiba, PR, Brazil, 2022

Regarding the number of AE per patient, an average of 1.47 AE per patient was observed in the pre-intervention period, 1.53 AE per patient in post-intervention I, and 1.43 AE per patient in post-intervention II, totaling 216 cases (Figure 1).

*pAE = Potential adverse event; ^†^AE = Adverse event

There was a reduction in the number of patients affected by two or more AE, with a variation from 27.8% (pre-intervention) to 11.3% (p = 0.002) in the post-intervention II period. Table 3 presents the AE according to their classification.

**Table 3 -:** Distribution of adverse events before and after the implementation of surgical checklists according to classification, degree of harm and avoidability. Curitiba, PR, Brazil, 2022

**Variable**	**Period**						**Total (n= 216)**	
	Pre-intervention (n= 91)		Post- intervention I (n= 75)		Post- intervention II (n= 50)			
	n	%	n	%	n	%	n	%
**Classification of adverse event**								
Medication/intravenous fluids	41	45	21	28	7	14	69	31.9
Clinical process/procedure	32	35.2	36	48	29	58	97	44.9
Medical devices/equipment	8	8.8	11	14.7	6	12	25	11.6
Healthcare-associated infection	7	7.7	4	5.4	5	10	16	7.4
Clinical administration	2	2.2	1	1.3	1	2	4	1.9
Patient accidents	1	1.1	1	1.3	0	0	2	0.9
Blood/blood products	0	0	0	0	2	4	2	0.9
Infrastructure/location/facilities	0	0	1	1.3	0	0	1	0.5
**Degree of harm**								
Mild	62	68.1	60	80	35	70	157	72.7
Moderate	24	26.4	15	20	10	20	49	22.7
Severe	4	4.4	0	0	5	10	9	4.2
Death	1	1.1	0	0	0	0	1	0.4
**Potential for avoidabilit** y								
Strongly avoidable	10	11	20	26.7	15	30	45	20.8
Potentially avoidable	72	79.1	47	62.7	29	58	148	68.5
Potentially non-avoidable	9	9.9	8	10.7	4	8	21	9.7
Strongly non-avoidable	0	0	0	0	2	4	2	0.1

The classification of the types AE that occurred before and after the interventions is presented in Table 4. A significant reduction in the prevalence of patients affected by urinary retention (33%; 14.4%; 3.1%, p < 0.001) and hemorrhage (9.3%; 7.2%; 0%, p = 0.012) was observed between the pre-intervention period and post-interventions I and II.

The opposite occurred for cases of skin lesions (due to surgical positioning/device/medical adhesive), which increased from 2.1% (pre-intervention) to 10.3% (post-interventions) (p = 0.043). No significant difference was found in the prevalence of patients with surgical wound infections (6.2%; 4.1%; 5.2%, p = 0.810) and hematoma/seroma (5.2%; 9.3%; 8.3%, p = 0.528).

**Table 4 -:** Distribution of adverse events before and after the implementation of surgical checklists, according to type. Curitiba, PR, Brazil, 2022

**Type of adverse event**	**Intervention period**						**Total (n= 216)**	
	Pre-intervention (n= 91)		Post-intervention I (n= 75)		Post-intervention II (n= 50)			
	n	%	n	%	n	%	n	%
Urinary retention	32	35.2	14	%	3	6	49	22.7
Hematoma/seroma	5	5.5	9	12	8	16	22	10.2
Hemorrhage	9	9.9	7	9.3	0	0	16	7.4
Epidural catheter displacement	7	7.7	7	9.3	1	2	15	6.9
Wound infection	6	6.6	4	5.3	5	10	15	6.9
Mucosal/skin lesion	2	2.2	7	9.3	6	12	15	6.9
Nausea/vomiting	6	6.6	3	4	2	4	11	5.1
Surgical positioning injury	0	0	4	5.3	5	10	9	4.2
Deep vein thrombosis	3	3.3	2	2.7	2	4	7	3.2
Lack of material/equipment/bed	2	2.2	2	2.7	1	2	5	2.3
Acute pain	0	0	4	5.3	0	0	4	1.9
Fracture	3	3.3	1	1.3	0	0	4	1.9
Dislocation	1	1.1	0	0	3	6	4	1.9
Neuropraxia	1	1.1	3	4	0	0	4	1.9
Pruritus	0	0	3	4	1	2	4	1.9
Suture dehiscence	1	1.1	1	1.3	1	2	3	1.4
Lower limb length discrepancy	2	2.2	1	1.3	0	0	3	1.4
Anesthetic block failure	1	1.1	0	0	2	4	3	1.4
Equipment failure	1	1.1	0	0	2	4	3	1.4
Loosening of synthetic material	1	1.1	1	1.3	1	2	3	1.4
Pulmonary embolism	0	0	0	0	3	6	3	1.4
Hypotension	2	2.2	0	0	0	0	2	0.9
Dura mater perforation	1	1.1	1	1.3	0	0	2	0.9
Transfusion reaction	0	0	0	0	2	2	2	0.9
Cerebrovascular accident	1	1.1	0	0	0	0	1	0.5
Septic shock	1	1.1	0	0	0	0	1	0.5
Subcutaneous emphysema	1	1.1	0	0	0	0	1	0.5
Medication error	1	1.1	0	0	0	0	1	0.5
Fistula	0	0	0	0	1	2	1	0.5
Hypoglycemia	1	1.1	0	0	0	0	1	0.5
Hypoxia	0	0	0	0	1	2	1	0.5
Opioid intoxication	0	0	1	1.3	0	0	1	0.5

Regarding the frequency of readmissions due to AE, no significant difference was observed before and after the implementation of checklists (p = 0.092): pre-intervention (9.7%; n = 6), post-intervention I (8.2%; n = 4), and post-intervention II (22.9%; n = 8).

## Discussion

The reduction in the prevalence of AE among patients undergoing hip and knee arthroplasties requires an understanding of the various factors that contribute to increased healthcare and surgical risks, as well as the corresponding critical assessment by managers regarding the interventions outlined in the institutional strategic planning to promote safe practices and protect patients from surgical errors and harm. In this study, a significant decline was observed in the prevalence and number of AE per patient after the implementation of two surgical checklists. These results add to the evidence indicating that the use of surgical safety checklists has fostered safe and value-based practices^(28)^.

The favorable result presented in this research is potentially the result of the efforts of health professionals to gradually adapt, develop, and implement surgical checklists to meet the recommendations of the SSSL, as well as the participation of leadership in the progressive development of a patient safety culture, a factor that contributes to the visibility of risks and the adoption of preventive actions.

Satisfactory results from the implementation of the surgical checklist were also observed in a study conducted at a tertiary hospital in Australia, where the authors reported a significant reduction in the mortality rate from 1.2% to 0.92%^(29)^. Similarly, a research carried out in teaching hospitals in the United Kingdom showed a decrease in the prevalence of AE, from 16.9% to 11.2% (p = 0.01)^(30)^.

In Norway, researchers observed that the concurrent use of a checklist applied in the pre- and postoperative phases, along with the intraoperative checklist, significantly reduced AE, reoperations, and unplanned readmissions^(31)^. This finding partially differs from the results of the present study, which found no difference in readmissions resulting from these complications. It is worth highlighting that the positive effects of checklists are also evidenced in surgical services in low- and middle-income countries, such as Brazil, with a 44% reduction in the overall incidence of AE^(32)^, supporting the findings presented here.

In this study, the significant reduction in patients with urinary retention after the interventions can be primarily explained by the observed decrease in the use of epidural anesthesia and acute analgesia via epidural catheter in the postoperative phase. Another potentially related factor is adherence to evidence-based guidelines for preventing this condition, including the cautious use of intrathecal opioids and the combination of different analgesic drugs for acute pain management^(33-34)^, even starting in the preoperative period to reduce opioid consumption in the postoperative phase^(35)^.

It is reiterated that the adoption of checklists makes it possible to improve preoperative planning and anticipate critical events by identifying intrinsic and extrinsic factors that increase the clinical-surgical risk of urinary retention episodes^(36)^. This finding is relevant, since such an AE exacerbates issues related to the bladder muscle, compromising its functionality and increasing the risk of urinary infections, consequently requiring additional procedures and leading to successive increases in risks and hospital length of stay^(36-37)^.

Regarding the impacts of the checklist on hemorrhage prevention, divergences are observed in the literature when considering different perioperative scenarios. While a study conducted in Norway revealed a decrease in postoperative bleeding cases from 2.6% to 1.0% (p< 0.001), as well as in costs associated with blood component transfusions^(38)^, another investigation conducted at a tertiary care center in Australia found no difference in the occurrence of this AE^(29)^.

The significant difference found in this study regarding the number of patients exposed to this AE before and after the interventions may be related to improvements in patient assessment, the use of equipment, and the surgical techniques employed. It is recognized that the SSSL, in the immediate preoperative stage, aims to anticipate the possibility of blood loss and enable the planning of multimodal actions, such as the prior reservation of labile coagulation factors and blood components, with the objective of minimizing and treating this eventuality, as well as preventing severe AE^(39)^. Thus, considering that these are elective surgeries, the evaluation carried out by the surgical, anesthesiology, and nursing teams during the preoperative phase provides an opportunity for harm prevention.

It should also be considered that this is a teaching hospital, and as such, the residents’ limited experience in performing surgical techniques represents a risk factor for patients and a challenge for the coordinators and professionals working in the patient safety units. Direct supervision of trainees contributes to high-quality training and safe learning, promoting patient safety through the optimization of operative time in hip and knee arthroplasties. Furthermore, improvements related to postoperative nursing care management, such as the assessment and management of surgical dressings and drains, and the application of cryotherapy, may have contributed to the reduction of hemorrhagic events^(40)^. The institutional checklist, applied in the immediate postoperative period, includes the verification of bleeding signs, whose early detection helps prevent case aggravation^(19)^.

The implementation of both types of checklists did not affect the reduction of hospital length of stay, contrary to the literature^(29)^. It is inferred that the greater severity and complexity of the patients may have contributed to the maintenance of the duration of the anesthetic-surgical process and hospital stay. Moreover, an increase in the prevalence of patients with skin tissue damage was observed, characterized as injuries from surgical positioning and/or injuries caused by medical devices or adhesives. Different factors may have contributed to the occurrence of these events in the studied sample, which could be explored in subsequent research. However, reducing the operative time^(41)^ and using appropriate devices and support surfaces^(42-43)^ are protective factors against these adverse events and should be addressed from a management perspective, in light of professional training and practice, with emphasis on the nursing team.

On the other hand, it is worth noting that, prior to the interventions, these adverse events may have been perceived by the healthcare team as common occurrences in the surgical field, consequently receiving less importance in terms of registration and reporting compared to other AE. The rise of aspects related to risk management and the improvement of protocols for the prevention of surgical incidents potentially stimulated the maturation of a patient safety culture over the course of the study. This development may have contributed to improvements in work processes, increasing the professionals’ awareness and commitment to identifying and recording these AE. As a result, it became possible to detect, through document tracking, events that had not previously been recorded in patient charts.

No significant decrease was identified in the prevalence of patients with surgical wound infections and hematomas/seromas, contrary to what was observed in hospitals in Ethiopia, where the prevalence of SSI dropped from 27.3% to 9.1% (p= 0.086)^(44)^, and in a research conducted in India, where a significant reduction in this type of AE was observed before and after the implementation of the checklist (14% to 9.5%)^(45)^. However, it is recognized that the use of these tools, in isolation, has a limited effect as a measure for preventing SSI^(46)^, as their occurrence is multifactorial, involving factors related to the environment, patient and surgical procedure^(47)^.

Nevertheless, it is noted that the prevalence of AE remains high in the hospital of the study, with the majority of these events being preventable, according to a cohort multicenter study involving 24 hospitals in Sweden, which reported a prevalence of AE in hip arthroplasties of 58.7%, with 76% of the records classified as preventable^(48)^. In Brazil, epidemiological studies evaluating the severity and preventability of these issues, exclusively in the orthopedic field, are incipient. However, it is observed that the incidence of AE of 33.7% was evidenced in a teaching hospital located in the southeastern region of Brazil, with more than half of the cases being preventable^(49)^.

The results presented in this research contribute to highlighting the potential role of implementing checklists in reducing AE and reiterate the importance of adopting good care practices and their monitoring through epidemiological studies. Another valuable aspect of this research for scientific knowledge is related to the AE tracking methodology used, which is replicable in different cultures and healthcare contexts. Active identification of AE helps to recognize and classify unfavorable outcomes, build quality indicators, and support the planning and revision of care protocols.

Furthermore, the results contribute to encouraging other researchers to investigate the topic in different settings, as the use of these tools is strongly influenced by the particularities of each healthcare unit, considering technological, demographic, epidemiological aspects, and the management models of healthcare organizations, in addition to the multidisciplinary team’s adherence to quality principles.

Among the limitations of this study, the retrospective analysis based on document review stands out, as it depends on the quality of the records, the sample size, and the fact that the study was conducted in a single hospital. Furthermore, it is acknowledged that multiple factors may interfere with healthcare quality and the occurrence of AE, and are not restricted to the interventions studied here.

## Conclusion

There was a reduction in the frequency and overall prevalence of AE after the implementation of surgical checklists, especially in the reduction of urinary retention and hemorrhage. However, an increase in the prevalence of skin lesions was observed. No effects were observed regarding the occurrence of surgical wound infections and hematomas/seromas, nor in hospital length of stay and readmissions due to AE.

## References

[1] Nyberg A., Olofsson B., Otten V., Haney M., Fagerdahl A. M. (2021). Patient safety during joint replacement surgery: experiences of operating room nurses. BMJ Open Qual.

[2] Barach P., Wiggin H., Risner P., Johnson J., Patrishkoff D., Kurra S., Sanchez J. A., Higgins R. S. D., Kent P. S. (2024). A perioperative safety and quality change management model and case study: Muda Health.

[3] Barneschi G., Raspanti F., Capanna R., Donaldson L., Ricciardi W., Sheridan S., Tartaglia R. (2021). Patient Safety in Orthopedics and Traumatology.

[4] DeMaio E. L., Marra G., Suleiman L. I., Tjong V. K. (2024). Global Health Inequities in Orthopaedic Care: Perspectives Beyond the US. Curr Rev Musculoskelet Med.

[5] Sauder N., Emara A. K., Rullán P. J., Molloy R. M., Krebs V. E., Piuzzi N. S. (2023). Hip and Knee Are the Most Litigated Orthopaedic Cases: A Nationwide 5-Year Analysis of Medical Malpractice Claims. J Arthroplasty.

[6] World Health Organization. (2009). The Conceptual Framework for the International Classification for Patient Safety [Internet].

[7] World Health Organization. (2021). Global Patient Safety Action Plan 2021-2030: towards eliminating avoidable harm in health care [Internet].

[8] Liu J., Liu P., Gong X., Liang F. (2020). Relating Medical Errors to Medical Specialties: A Mixed Analysis Based on Litigation Documents and Qualitative Data. Risk Manag Healthc Policy.

[9] Hafez A. T., Omar I., Purushothaman B., Michla Y., Mahawar K. (2022). Never events in orthopedics: A nationwide data analysis and guidance on preventative measures. Int J Risk Saf Med.

[10] Tan J., Ross J. M., Wright D., Pimentel M. P. T., Urman R. D. (2023). A Contemporary Analysis of Closed Claims Related to Wrong-Site Surgery. Jt Comm J Qual Patient Saf.

[11] Gatfield S. A., Atkinson K. V., Fountain D., Machin J. T., Navaratnam A. V., Hutton M. (2023). Getting it right first time: national survey of surgical site infection 2019. Ann R Coll Surg Engl.

[12] Stefani L., Borges P. K. O., Gaspar M. D. R. (2022). Surgical site infections: surgical reoperation and infection in clean and potentially contaminated surgeries. Rev Enferm UFSM.

[13] Kumar M., Kumar R., Kumar S. (2021). Coatings on orthopedic implants to overcome present problems and challenges: A focused review. Mater Today Proc.

[14] The Joint Commission. (2023). Overcoming challenges in orthopedic care: Through education and standardization [Internet].

[15] World Health Organization. (2009). WHO guidelines for safe surgery: Safe surgery saves lives [Internet].

[16] Venneri F., Brown L. B., Cammelli F., Haut E. R., Donaldson L., Ricciardi W., Sheridan S., Tartaglia R. (2021). Safe Surgery Saves Lives.

[17] World Health Organization. (2017). Patient safety making health care safer [Internet].

[18] Maziero E. C. S., Silva A. E. B. C., Mantovani M. F., Cruz E. D. A. (2015). Adherence to the use of the surgical checklist for patient safety. Rev Gaucha Enferm.

[19] Alpendre F. T., Cruz E. D. A., Dyniewicz A. M., Mantovani M. F., Silva A. E. B. C., Santos G. S. (2017). Safe surgery: validation of pre and postoperative checklists. Rev. Latino-Am. Enfermagem.

[20] Champagne F., Contandriopoulos A. P., Brousselle A., Hartz Z., Denis J. L., Brousselle A., Champagne F., Contandriopoulos A. P., Hartz Z. (2021). A avaliação no campo da saúde: conceitos e métodos.

[21] Baker G. R., Norton P. G., Flintoft V., Blais R., Brown A., Cox J. (2004). The Canadian Adverse Events Study: the incidence of adverse events among hospital patients in Canada. CMAJ.

[22] Mendes W., Travassos C., Martins M., Marques P. M. (2008). Adjustment of adverse events assessment forms for use in Brazilian hospitals. Rev Bras Epidemiol.

[23] Griffin F. A., Resar R. K. (2009). IHI global trigger tool for measuring adverse events. IHI innovation series white paper [Internet].

[24] Alpendre F. T., Cruz E. D. A., Batista J., Maziero E. C. S., Brandão M. B. (2022). Translation, cross-cultural adaptation and content validation of the Global Trigger Tool surgical module. Rev Bras Enferm.

[25] Centers for Disease Control and Prevention; National Healthcare Safety Network. (2024). Surgical site infection event (SSI) [Internet].

[26] Mendes W., Martins M., Rozenfeld S., Travassos C. (2009). The assessment of adverse events in hospitals in Brazil. Int J Qual Health Care.

[27] Batista J., Cruz E. D. A., Alpendre F. T., Rocha D. J. M., Brandão M. B., Maziero E. C. S. (2019). Prevalence and avoidability of surgical adverse events in a teaching hospital in Brazil. Rev. Latino-Am. Enfermagem.

[28] González-Mariño M. A. (2024). Safety of surgery: quality assessment of meta-analyses on the Who checklist. Ann Med Surg (Lond).

[29] Jager E., Gunnarsson R., Ho Y. H. (2019). Implementation of the World Health Organization Surgical Safety Checklist Correlates with Reduced Surgical Mortality and Length of Hospital Admission in a High-Income Country. World J Surg.

[30] Mayer E. K., Sevdalis N., Rout S., Caris J., Russ S., Mansell J. (2016). Surgical Checklist Implementation Project: The Impact of Variable WHO Checklist Compliance on Risk-adjusted Clinical Outcomes After National Implementation: A Longitudinal Study. Ann Surg.

[31] Storesund A., Haugen A. S., Flaatten H., Nortvedt M. W., Eide G. E., Boermeester M. A. (2020). Clinical Efficacy of Combined Surgical Patient Safety System and the World Health Organization’s Checklists in Surgery: A Nonrandomized Clinical Trial. JAMA Surg.

[32] White M. C., Peven K., Clancy O., Okonkwo I., Bakolis I., Russ S. (2021). Implementation Strategies and the Uptake of the World Health Organization Surgical Safety Checklist in Low and Middle Income Countries: A Systematic Review and Meta-analysis. Ann Surg.

[33] Cosgrave D., Shanahan E., Conlon N. (2017). Intrathecal opioids [Internet].

[34] Eldh A. C., Joelsson-Alm E., Wretenberg P., Hälleberg-Nyman M. (2021). Onset Prevention of urinary retention in Orthopaedic Nursing and rehabilitation, OPTION-a study protocol for a randomised trial by a multi-professional facilitator team and their first-line managers’ implementation strategy. Implement Sci.

[35] Passias B. J., Johnson D. B., Schuette H. B., Secic M., Heilbronner B., Hyland S. J. (2023). Preemptive multimodal analgesia and post-operative pain outcomes in total hip and total knee arthroplasty. Arch Orthop Trauma Surg.

[36] Cha Y. H., Lee Y. K., Won S. H., Park J. W., Ha Y. C., Koo K. H. (2020). Urinary retention after total joint arthroplasty of hip and knee: Systematic review. J Orthop Surg (Hong Kong).

[37] Souza I. T., Fernandes  J., Rangel P. I., Dias L. V. R., Magalhaes S. S., Teixeira G. C. (2021). Retenção urinária pós-operatória, prevenção e tratamento: um relato de caso. Rev Urominas [Internet].

[38] Haugen A. S., Waehle H. V., Almeland S. K., Harthug S., Sevdalis N., Eide G. E. (2019). Causal Analysis of World Health Organization’s Surgical Safety Checklist Implementation Quality and Impact on Care Processes and Patient Outcomes: Secondary Analysis From a Large Stepped Wedge Cluster Randomized Controlled Trial in Norway. Ann Surg.

[39] Shah A., Palmer A. J. R., Klein A. A. (2020). Strategies to minimize intraoperative blood loss during major surgery. Br J Surg.

[40] Tsinaslanidis G., Tsinaslanidis P., Mahajan R. H. (2020). Perioperative Pain Management in Patients Undergoing Total Hip Arthroplasty: Where Do We Currently Stand?.

[41] Li N., Cui D., Shan L., Li H., Feng X., Zeng H. (2023). The prediction model for intraoperatively acquired pressure injuries in orthopedics based on the new risk factors: a real-world prospective observational, cross-sectional study. Front Physiol.

[42] Santana L. O., Leal S. M. C., Trevilato D. D., Moraes C. M., Santos G. N. S. R., Treviso P. (2024). Nursing interventions for preventing pressure ulcers in the perioperative period. Rev SOBECC.

[43] Alcântara C. M. P., Oliveira E. L. S., Campanili T. C. G. F., Santos R. S. C. S., Santos V. L. C. G., Nogueira P. C. (2021). Prevalence and associated factors of medical adhesive-related skin injury in cardiac critical care units. Rev Esc Enferm USP.

[44] Sibhatu M. K., Taye D. B., Gebreegziabher S. B., Mesfn E., Bashir H. M., Varallo J. (2022). Compliance with the World Health Organization’s surgical safety checklist and related postoperative outcomes: a nationwide survey among 172 health facilities in Ethiopia. Patient Saf Surg.

[45] Raj V., Agarwal S., Gupta A. K., Sharma V. (2024). Application of the “who surgical safety” checklist and analysis of its impact on postoperative results in emergencies surgeries: a prospective observation study. Int J Acad Med Pharm [Internet].

[46] Ching P. R. (2024). Care Bundles in Surgical Site Infection Prevention: A Narrative Review. Curr Infect Dis Rep.

[47] Sikora A., Zahra F. (2023). Nosocomial Infections.

[48] Magnéli M., Kelly-Pettersson P., Rogmark C., Gordon M., Sköldenberg O., Unbeck M. (2023). Timing of adverse events in patients undergoing acute and elective hip arthroplasty surgery: a multicentre cohort study using the Global Trigger Tool. BMJ Open.

[49] Zanetti A. C. B., Dias B. M., Bernardes A., Capucho H. C., Balsanelli A. P., Moura A. A. (2021). Incidence and preventability of adverse events in adult patients admitted to a Brazilian teaching hospital. PLoS One.

