# Supplementary material for: Prevalence of adverse events in hip and knee arthroplasties following the implementation of surgical checklists
Source: Rev Lat Am Enfermagem. 2025 May 19;33:e4557. doi: 10.1590/1518-8345.7658.4557 (PMC12088655; doi:10.1590/1518-8345.7658.4557)
Supplement: Translation

# Translation: Prevalência de eventos adversos em artroplastias de quadril e joelho após aplicação de *checklists* cirúrgicos*

**DOI:** 10.1590/1518-8345.7658.4558

**Authors:** Josemar Batista, Elaine Drehmer de Almeida Cruz

**Affiliations:** 1 Universidade Federal do Paraná, Departamento de Enfermagem, Curitiba, PR, Brasil; 2 Universidade Positivo, Escola de Ciências da Saúde, Curitiba, PR, Brasil

**Keywords:** Assistência Perioperatória, Erros Médicos, Segurança do Paciente, Artroplastia, Lista de Checagem, Avaliação de Processos e Resultados em Cuidados de Saúde

## Abstract

identificar a prevalência de eventos adversos em pacientes submetidos a artroplastias de quadril e joelho após a implementação de *checklists* cirúrgicos.

pesquisa avaliativa, do tipo análise dos efeitos, conduzida em três períodos: antes (0- 2010) e após a intervenção (I- 2013; II- 2016), com consulta retrospectiva em uma amostra aleatória simples de 291 prontuários, entre novembro de 2020 e março de 2022. Utilizaram-se os formulários do *Canadian Adverse Events Study* e *Global Trigger Tool* para rastrear e confirmar eventos adversos. Os casos foram analisados por estatística descritiva e inferencial; valores de p ≤ 0,05 indicaram significância estatística.

observou-se, nos períodos pós aplicação de *checklists* cirúrgicos, uma redução na frequência de pacientes acometidos por dois ou mais eventos, de 27,8% para 11,3% (p = 0,002), e na prevalência geral, de 63,9% para 36,1% (p < 0,001). Houve uma redução na prevalência de pacientes acometidos por retenção urinária (33% para 3,1%; p < 0,001) e hemorragia (9,3% para 0%; p = 0,012). Registrou-se um aumento na prevalência de lesões de pele, de 2,1% para 10,3% (p = 0,043).

houve uma redução na prevalência geral e da frequência de eventos adversos em pacientes submetidos à artroplastia após a implementação de *checklists*.

## Destaques

**(1)** Elevada prevalência de eventos adversos em artroplastias de quadril e joelho. **(2)** *Checklists* cirúrgicos reduzem a prevalência de eventos adversos em artroplastias.**(3)** Houve uma diminuição significativa de pacientes acometidos por dois ou mais eventos adversos. **(4)** Investigar o impacto das intervenções contribui para a promoção de uma cultura de segurança. **(5)** Resultados são relevantes para fortalecer ações básicas de segurança perioperatória.

## Introdução

As evidências de que a mortalidade cirúrgica é superior entre aqueles que recebem cuidados de menor qualidade, quando comparados aos que não têm acesso aos serviços cirúrgicos, reiteram a importância de ações inovadoras direcionadas à oferta de melhores práticas no contexto perioperatório^(1-2)^. Esse dado reforça o nível de criticidade do setor, tanto nas exigências orientadas para as tarefas e processos quanto nas demandas cognitivas dos profissionais de saúde, visando à oferta de cuidados de excelência^(2)^.

É reconhecido que a complexidade da área cirúrgica contribui para a ocorrência de erros e incidentes relacionados à assistência, especialmente em intervenções que exigem o uso de ferramentas e implantes, como é o caso das artroplastias de joelho e quadril^(3-5)^. Conceitualmente, o erro consiste na falha em executar uma ação planejada conforme o pretendido ou em aplicar um plano de forma incorreta. Essas ocorrências aumentam o risco de incidentes com danos ao paciente, definidos como eventos adversos (EA)^(6-7)^.

Na assistência ortopédica, independentemente do tipo de anestesia e do procedimento cirúrgico ao qual o paciente é submetido, erros não são raros. Há consenso de que essa especialidade cirúrgica apresenta uma probabilidade superior de danos evitáveis quando comparada a outras^(3,8-9)^. Existe maior chance de ocorrência de cirurgias em local errado^(10)^ e de eventos infecciosos graves, com necessidade de reintervenção operatória^(11-12)^, principalmente devido às particularidades inerentes ao implante de materiais^(13)^. A falta de padronização em equipamentos, procedimentos e protocolos nessa especialidade cirúrgica soma-se aos riscos e potencializa a ocorrência de danos^(14)^.

Diante dos distintos fatores críticos que contribuem para erros e EA cirúrgicos em geral, a Organização Mundial da Saúde lançou, no biênio 2007-2008, o Programa Cirurgias Seguras Salvam Vidas (PCSSV), recomendando a utilização de *checklists* cirúrgicos e sua adaptação opcional para atender demandas loco-regionais. O instrumento, em sua versão original, contém 19 itens e tem como objetivo garantir a segurança do paciente^(15-16)^. Desde então, pesquisas evidenciam os benefícios dessa ferramenta, impactando na redução de 33% de EA^(16-17)^, com resultados que reforçam sua importância na promoção da qualidade assistencial e na prevenção de erros.

Nesse contexto, as ações de segurança destinadas a atender aos objetivos do referido programa foram adotadas de forma gradual no hospital desta pesquisa. Em 2011, o *checklist* cirúrgico recomendado pela OMS foi adaptado e pioneiramente utilizado pela especialidade de ortopedia em cirurgias com implante^(18)^. No segundo semestre de 2014, outro *checklist* foi disponibilizado para aplicação na unidade de internação ortopédica, voltado à verificação de itens de segurança relacionados às fases pré e pós-operatórias^(19)^.

Ao analisar os recursos humanos e financeiros empenhados na área cirúrgica, bem como as adaptações e/ou elaboração de itens de checagem para a consolidação de ações e políticas institucionais no hospital em estudo, considerou-se importante a realização de estudos avaliativos. Esses têm por finalidade julgar os efeitos da implementação dessas ferramentas no contexto assistencial, por meio da análise pré e pós-intervenção.

Assim, o objetivo desta pesquisa foi identificar a prevalência de eventos adversos em pacientes submetidos a artroplastias de quadril e joelho após a implementação de *checklists* cirúrgicos.

## Método

### Delineamento do estudo

Trata-se de uma pesquisa avaliativa do tipo análise de efeitos^(20)^, documental, retrospectiva e com abordagem quantitativa.

### Cenário do estudo

A pesquisa foi realizada em um hospital de ensino de grande porte na região sul do Brasil, que atende exclusivamente pelo Sistema Único de Saúde. A especialidade cirúrgica envolvida foi a ortopedia, e foram considerados os dados de internações de pacientes submetidos a artroplastias de quadril e joelho em períodos distintos, correspondentes a antes e após a implementação de duas modalidades de *checklists* cirúrgicos. Esses períodos são aqui denominados pré-intervenção e pós-intervenções I e II. A escolha dessa população, especificamente, justifica-se pela complexidade cirúrgica do procedimento com implante de prótese e pelo pioneirismo da especialidade no uso desses instrumentos.

A Intervenção I ocorreu em 2011 com a aplicação de um *checklist* para uso no centro cirúrgico, composto por 45 itens organizados em quatro momentos: (I) recepção do paciente, (II) antes da indução anestésica, (III) antes da incisão cirúrgica, e (IV) antes de o paciente deixar a sala operatória^(18)^. Em 2014, foi implementado um *checklist* para uso na unidade de internação ortopédica (Intervenção II), com 97 itens de segurança distribuídos em seis momentos: (1) Identificação; (2) Pré-operatório: itens a serem verificados antes de o paciente ser encaminhado ao centro cirúrgico; (3) Pós-operatório Imediato: verificação de indicadores de segurança nas primeiras 24 horas após o procedimento anestésico-cirúrgico; (4) Pós-operatório Mediato: itens relacionados à avaliação da dor, dos aspectos da ferida operatória e do restabelecimento dos sistemas fisiológicos; (5) Complicações: registro de complicações pós-operatórias, como as infecciosas; e (6) Alta hospitalar/transferência: itens de segurança relacionados ao estado geral do paciente, às condições do sítio cirúrgico e de dispositivos, além de orientações para cuidados domiciliares e retorno ambulatorial^(19)^.

Ambos os *checklists* incluem a verificação de elementos que contribuem para a prevenção de erros e a detecção de não conformidades relacionadas à segurança e à qualidade assistencial. Adotou-se um intervalo de dois anos para investigação e análise, correspondendo ao ano de 2010 (pré-intervenção) e aos anos de 2013 e 2016 (pós-intervenção I e II).

### População e critérios de seleção

A fonte de informações de interesse para o estudo foi o banco de dados disponibilizado pelo serviço de informática do hospital, composto pela totalidade das cirurgias de artroplastias de quadril e joelho realizadas no período de 1 de janeiro a 31 dezembro de 2010 (pré-intervenção), 1 de janeiro a 31 dezembro de 2013 (pós-intervenção I) e 1 de janeiro a 31 dezembro de 2016 (pós-intervenção II). Os registros de prontuários repetidos foram eliminados, mantendo-se apenas aqueles referentes à primeira cirurgia realizada durante a internação analisada, correspondente à internação índice.

Foram incluídos os prontuários de pacientes adultos e com tempo de internação mínimo de 24 horas. Casos de óbitos ocorridos no intraoperatório e com período de internação inferior a 24 horas também foram incluídos. Casos com diagnóstico relacionado a doenças psiquiátricas foram excluídos, conforme previamente estabelecido^(21-22)^.

### Definição da amostra

Para estimar o tamanho amostral, foi realizado um estudo piloto para detectar diferenças significativas na prevalência de EA ao comparar os períodos pré-intervenção e pós-intervenção II. A estimativa inicial de prevalência foi obtida a partir de uma amostra piloto com 25 prontuários selecionados aleatoriamente para cada condição, na qual obteve-se uma prevalência de 60% e 40% de EA, respectivamente, aplicando-se a metodologia de coleta de dados descrita a seguir. Para o cálculo amostral, considerou-se um nível de significância de 5% e um poder do teste de 80%, resultando em um tamanho mínimo de amostra de 97 prontuários para cada condição.

A seleção aleatória simples dos prontuários elegíveis foi realizada a partir de uma lista geral de cirurgias gerada pelo banco de dados institucional, utilizando o *software Microsoft Office Excel* 2016^®^. Os prontuários indisponíveis no serviço de arquivamento físico foram substituídos pelos prontuários imediatamente subsequentes na referida lista, sem perdas insubstituíveis. Os prontuários utilizados no estudo-piloto foram incluídos na pesquisa.

### Coleta de dados e instrumentos utilizados

A coleta de dados foi realizada entre os meses de novembro de 2020 e março de 2022, por meio de consulta retrospectiva exclusivamente das informações contidas nos prontuários físicos, e sistematizada em duas fases, conforme a metodologia adaptada do protocolo do *Canadian Adverse Events Study* ^(21)^. A Fase I (revisão primária) foi conduzida por um único enfermeiro e pesquisador principal, consistindo na busca e identificação de potenciais EA (pEA), orientada por dois formulários de rastreamento.

O primeiro formulário corresponde ao protocolo do *Canadian Adverse Events Study*, composto por 17 critérios explícitos de rastreamento de pEA relacionados à cirurgia, anestesia, medicamentos, diagnóstico, cuidados e tratamentos não medicamentosos^(21)^. Esse protocolo foi traduzido e adaptado para uso no Brasil^(22)^. O segundo formulário de rastreamento refere-se ao módulo cirúrgico do *Global Trigger Tool*, desenvolvido pelo *Institute for Healthcare Improvement* dos Estados Unidos da América. Ele foi aplicado com o objetivo de ampliar a busca por rastreadores de EA, sendo composto por 11 gatilhos para a identificação de pEA ocorridos nos períodos intra e pós-operatórios^(23)^, traduzidos e adaptados transculturalmente para o contexto brasileiro^(24)^.

Para a identificação de pEA ocorridos após alta-hospitalar, utilizaram-se os registros contidos nas fichas de consultas ambulatoriais que compõem os prontuários dos pacientes. Nos casos de Infecção de Sítio Cirúrgico (ISC), foram adotados os critérios dos *Centers for Disease Control and Prevention* que definem a infecção como aquela que ocorre em até 30 dias e/ou 90 dias após um procedimento cirúrgico com inserção de implante^(25)^.

Nos casos em que foi identificado ao menos um critério de rastreamento, preencheu-se o formulário de avaliação de pEA^(21-22)^ e o roteiro semiestruturado para caracterização do perfil demográfico, clínico, cirúrgico e anestésico, elaborado para a pesquisa.

Os registros da Fase I (revisão primária) foram analisados na Fase II (revisão secundária), correspondente à etapa de confirmação ou descarte do EA, mediante consenso de um comitê de especialistas^(21-22)^. Esse comitê foi formado, de modo intencional, pelo pesquisador principal, um enfermeiro e um médico, todos com titulação de mestre e com experiência superior a 10 anos na área de gestão da qualidade e segurança do paciente.

Para julgar os pEA, utilizaram-se os formulários preenchidos na etapa anterior. Para confirmar ou descartar o caso, adotou-se o conceito de EA proposto pela OMS^(6-7)^, e foram utilizadas três escalas. A primeira avaliou a gravidade do EA^(6)^. A segunda e a terceira escalas foram aplicadas para determinar, respectivamente, se o dano foi causado pela assistência prestada ao paciente e o grau de evitabilidade do evento. Essas escalas são compostas por seis escores, variando de (1) “praticamente nenhuma evidência” a (6) “evidência praticamente certa”. Consideraram-se EA evitáveis aqueles com escores ≥ 4 pontos^(21,26)^.

### Variáveis do estudo

As variáveis quantitativas e/ou categóricas registradas foram: sexo, idade (em anos), classificação da cirurgia (eletiva; emergência), potencial de contaminação (limpa; potencialmente contaminada; contaminada; infectada), tipo de anestesia (inalatória/sedação; geral; raquidiana; peridural; bloqueio; local), e risco cirúrgico de acordo com classificação da *American Society of Anesthesiologists* – ASA. Além disso, foram consideradas as comorbidades (fatores de risco intrínsecos) informadas na ficha de avaliação pré-anestésica e o uso de dispositivos médico-hospitalares (fatores de risco extrínsecos). Também foram extraídas informações para mensurar o tempo de internamento do paciente no pré-operatório (< 24 horas; ≥ 24 horas) e o tempo de permanência hospitalar (em dias).

Os EA foram classificados em: (1) leve; (2) moderado; (3) grave; e (4) óbito^(6)^. O grau de evitabilidade dos EA foi definido com a seguinte a subclassificação: (A) Fortemente evitável (escore 6); (B) Potencialmente evitável (escores 4 e 5); (C) Potencialmente não evitável (escores 2 e 3); e (D) Fortemente não evitável (escore 1)^(27)^.

Os EA confirmados também foram categorizados segundo a Classificação Internacional para a Segurança do Paciente na classe 1 (tipo de incidente), distribuídos nas seguintes categorias: (A) Administração clínica; (B) Processo/procedimento clínico; (C) Documentação; (D) Infecção relacionada à assistência à saúde (IRAS); (E) Medicação/fluidos endovenosos; (F) Sangue/hemoderivados; (G) Nutrição; (H) Oxigênio/gases/vapores; (I) Dispositivos/equipamentos médicos; (J) Comportamento; (K) Acidentes com o paciente; (L) Infraestrutura/local/instalações; e (M) Recursos/gestão organizacional^(6)^.

### Tratamento e análise dos dados

Os dados coletados foram inseridos, por dupla digitação, em uma planilha do *Microsoft Office Excel* 2016^®^. Após a checagem e a correção de inconsistências, foram analisados com assessoria estatística e utilizando o *software International Business Machines Corporation*™ - *Statistical Package for the Social Sciences* (IBM™ - SPSS) versão 20.0 para *Windows*.

As medidas utilizadas foram: prevalência de EA entre os pacientes: [(número de pacientes com pelo menos um EA/número total de pacientes) x 100]; e proporção de EA evitáveis [(número de EA evitáveis/número total de EA) x 100].

As variáveis quantitativas foram descritas por estatística descritiva univariada, enquanto as categóricas foram apresentadas por frequências absolutas e relativas. Para comparar os períodos pré-intervenção, intervenção I e intervenção II nas variáveis quantitativas, utilizou-se o modelo de análise da variância (ANOVA) com um fator ou o teste não-paramétrico de Kruskal-Wallis. Para comparações múltiplas, foi aplicado o teste *post-hoc* de Dunn, com os valores de p corrigidos pelo método de Bonferroni. A normalidade das variáveis quantitativas foi avaliada pelo teste de Kolmogorov-Smirnov. Valores de p ≤ 0,05 indicaram significância estatística.

### Aspectos éticos

A pesquisa foi aprovada pelo comitê de ética institucional sob o parecer número 3.651.686. Solicitou-se a dispensa da obtenção do Termo de Consentimento Livre e Esclarecido (TCLE) relativo aos pacientes. Os revisores/especialistas convidados a participar da Fase II formalizaram seu aceite mediante a assinatura do TCLE.

## Resultados

Na revisão primária dos 291 prontuários, constatou-se que a média de idade (respectivamente nas fases pré-intervenção, pós-intervenção I e pós-intervenção II) foi de 57,2 anos (desvio padrão ‒ DP ± 14,4), 56,5 (DP ± 15,5) e 59,5 (DP ± 15,4), sem diferença significativa entre os períodos analisados (p = 0,355). Quanto aos fatores de risco intrínsecos dos pacientes, observou-se que não houve diferença pré e pós-intervenções (p = 0,822). As demais características demográficas e clínicas estão apresentadas na Tabela 1.

**Tabela 1 -:** Perfil demográfico-clínico de pacientes submetidos a artroplastias de quadril e joelho antes e após a implementação de *checklists*cirúrgicos. Curitiba, PR, Brasil, 2022

**Variável**	**Pré-intervenção (n=97)**	**Pós-intervenção I (n=97)**	**Pós-intervenção II (n=97)**	**p-valor** *
	**n (%)**	**n (%)**	**n (%)**	
**Sexo**				
Feminino	56 (57,7)	59 (60,8)	62 (63,9)	0,677
Masculino	41 (42,3)	38 (39,2)	35 (36,1)	
**Comorbidade/fator de risco**				
Hipertensão arterial sistêmica				
Sim	58 (59,8)	50 (51,6)	56 (57,7)	0,484
Não	39 (40,2)	47 (48,5)	41 (42,3)	
Tabagismo				
Sim	15 (15,5)	25 (25,8)	13 (13,4)	0,057
Não	82 (84,5)	72 (74,2)	84 (86,6)	
Diabetes *mellitus*				
Sim	12 (12,4)	14 (14,4)	19 (19,6)	0,359
Não	85 (87,6)	83 (85,6)	78 (80,4)	
Pneumopatia ^†^				
Sim	10 (10,3)	6 (6,2)	6 (6,2)	0,455
Não	87 (89,7)	91 (93,8)	91 (93,8)	
Doenças de tireoide ^‡^				
Sim	9 (9,3)	11 (11,3)	7 (7,2)	0,613
Não	88 (90,7)	86 (88,7)	90 (92,8)	
Artrite reumatoide				
Sim	6 (6,2)	3 (3,1)	3 (3,1)	0,457
Não	91 (93,8)	94 (96,9)	94 (96,9)	
Cardiopatia				
Sim	6 (6,2)	7 (7,2)	7 (7,2)	0,948
Não	91 (93,8)	90 (92,8)	90 (92,8)	
Hepatite				
Sim	5 (5,2)	7 (7,2)	4 (4,1)	0,629
Não	92 (94,9)	90 (92,8)	93 (95,9)	
Dislipidemia				
Sim	4 (4,1)	2 (2,1)	5 (5,2)	0,516
Não	93 (95,9)	95 (97,9)	92 (94,9)	
Etilismo				
Sim	4 (4,1)	4 (4,1)	6 (6,2)	0,741
Não	93 (95,9)	93 (95,9)	91 (93,8)	
Hemofilia				
Sim	3 (3,1)	10 (10,3)	8 (8,3)	0,135
Não	94 (96,9)	87 (89,7)	89 (91,8)	
Neoplasia				
Sim	2 (2,1)	2 (2,1)	1 (1)	-
Não	95 (97,9)	95 (97,9)	96 (99)	
Osteoporose				
Sim	2 (2,1)	2 (2,1)	0 (0)	-
Não	95 (97,9)	95 (97,9)	97 (100)	

*Teste de Qui-quadrado, p< 0,05; ^†^Pneumopatia = Asma, bronquite, edema agudo de pulmão, enfisema pulmonar, doença pulmonar obstrutiva crônica; ^‡^Doenças de tireoide = Hipotireoidismo, hipertireoidismo

O tempo médio de hospitalização foi de 3,7 dias (DP ± 1,8) em 2010 (pré-intervenção), 4,9 dias (DP ± 3,7) em 2013 (pós-intervenção I) e 4,6 dias (DP ± 4,4), em 2016 (pós-intervenção II), com diferença significativa entre os períodos (p = 0,008), destacando-se entre 2010 e 2013 (p = 0,006).

Verificou-se, durante a internação em todos os períodos analisados, a presença de fatores de riscos extrínsecos, com destaque para o uso de cateter venoso periférico (100% em todos os períodos) e cateter peridural (88,7%; 71,1% e 4,1%, respectivamente). Este último apresentou uma redução significativa (p < 0,001) no uso antes e após as intervenções I e II, respectivamente. Houve redução significativa de pacientes submetidos à anestesia peridural (p < 0,001) e sedação (p = 0,008); o oposto ocorreu para a anestesia geral (p = 0,001), conforme demonstrado na Tabela 2, juntamente com outras características de interesse.

**Tabela 2 -:** Distribuição do perfil cirúrgico-anestésico e de internação de pacientes submetidos à artroplastias de quadril e joelho antes e após a implementação de *checklists*cirúrgicos. Curitiba, PR, Brasil, 2022

**Variável**	**Pré-intervenção (n=97)**	**Pós-intervenção I (n=97)**	**Pós-intervenção II (n=97)**	p-valor*
	**n (%)**	**n (%)**	**n (%)**	
**Diagnóstico pré-operatório**				-
Gonartrose	36 (37,1)	37 (38,1)	46 (47,4)	
Coxartrose	32 (33)	34 (35,1)	36 (37,1)	
Soltura asséptica de componente	12 (12,4)	8 (8,3)	8 (8,3)	
Coxartrose secundária	7 (7,2)	3 (3,1)	2 (2,1)	
Gonartrose secundária	4 (4,1)	11 (11,3)	3 (3,1)	
Outro ^†^	6 (6,2)	4 (4,1)	2 (2)	
**Tempo de internação pré-operatório** 0,343				
< 24 horas	91 (93,8)	88 (90,7)	93 (95,9)	
≥ 24 horas	6 (6,2)	9 (9,3)	4 (4,1)	
**Procedimento cirúrgico**				0,220
Artroplastia total de quadril	44 (45,4)	43 (44,3)	36 (37,1)	
Artroplastia total de joelho	37 (38,1)	45 (46,4)	51 (52,6)	
Artroplastia de revisão de quadril	9 (9,3)	8 (8,3)	6 (6,2)	
Artroplastia de revisão de joelho	7 (7,2)	1 (1)	4 (4,1)	
**Classificação da cirurgia**				-
Eletiva	95 (97,9)	97 (100)	97 (100)	
Emergência	2 (2,1)	0 (0)	0 (0)	
**Potencial de contaminação**				-
Limpa	97 (100)	97 (100)	96 (99)	
Infectada	0 (0)	0 (0)	1 (1)	
**Risco cirúrgico ASA** ^‡^				0,271
I	22 (22,7)	14 (14,4)	12 (12,4)	
II	61 (62,9)	72 (74,2)	70 (72,2)	
III	14 (14,4)	11 (11,3)	15 (15,5)	
**Tipo de anestesia** ^§^				
Peridural	86 (88,7)	65 (67)	7 (7,2)	< 0,001
Raquidiana	73 (75,3)	74 (76,3)	70 (72,2)	0,790
Sedação	71 (73,2)	61 (62,9)	50 (51,6)	0,008
Geral	21 (21,7)	23 (23,7)	43 (44,3)	0,001
Bloqueio	0 (0)	2 (2,1)	0 (0)	-
Local	0 (0)	1 (1)	4 (4,1)	-

*Teste de Qui-quadrado, p<0,05; ^†^Outro = Fratura de colo de fêmur, quebra de material de síntese, osteonecrose de fêmur, fratura periprotética, fratura de fêmur, artropatia hemofílica, infecção operatória; ^‡^ASA = *American Society of Anesthesiologists*; ^§^Um mesmo paciente pode ser submetido a mais de um tipo de anestesia

A prevalência de EA foi de 63,9%, 50,5% e 36,1%, respectivamente, para os períodos pré-intervenção, pós-intervenção I e pós-intervenção II, com redução significativa (p < 0,001). A detecção do EA ocorreu, predominantemente, durante a hospitalização, com diferença expressiva entre os períodos pré e pós-intervenções (p = 0,046).

Quanto ao número de EA por paciente, verificou-se uma média de 1,47 EA por paciente no período pré-intervenção, 1,53 EA por paciente no pós-intervenção I e 1,43 por paciente no pós-intervenção II, totalizando 216 casos (Figura 1).

**Figura 1 -:** Fluxograma da revisão dos prontuários e estimativa de prevalência de eventos adversos antes e após a implementação de *checklists*cirúrgicos. Curitiba, PR, Brasil, 2022

*pEA = Potencial evento adverso; ^†^EA = Evento adverso

Houve redução no número de pacientes acometidos por dois ou mais EA, com variação de 27,8% (pré-intervenção) para 11,3% (p = 0,002) no pós-intervenção II. A Tabela 3 apresenta os EA segundo sua classificação.

**Tabela 3 -:** Distribuição dos eventos adversos antes e após a implementação de *checklists*cirúrgicos segundo classificação, grau de dano e evitabilidade. Curitiba, PR, Brasil, 2022

**Variável**	**Período**						**Total (n= 216)**	
	Pré-intervenção (n= 91)		Pós-intervenção I (n= 75)		Pós-intervenção II (n= 50)			
	n	%	n	%	n	%		%
**Classificação do evento adverso**								
Medicação/fluidos endovenosos	41	45	21	28	7	14	69	31,9
Processo/procedimento clínico	32	35,2	36	48	29	58	97	44,9
Dispositivos/equipamento médicos	8	8,8	11	14,7	6	12	25	11,6
Infecção relacionada à assistência à saúde	7	7,7	4	5,4	5	10	16	7,4
Administração clínica	2	2,2	1	1,3	1	2	4	1,9
Acidentes com o paciente	1	1,1	1	1,3	0	0	2	0,9
Sangue/hemoderivados	0	0	0	0	2	4	2	0,9
Infraestrutura/local/instalações	0	0	1	1,3	0	0	1	0,5
**Grau de dano**								
Leve	62	68,1	60	80	35	70	157	72,7
Moderado	24	26,4	15	20	10	20	49	22,7
Grave	4	4,4	0	0	5	10	9	4,2
Óbito	1	1,1	0	0	0	0	1	0,4
**Potencial de evitabilidade**								
Fortemente evitável	10	11	20	26,7	15	30	45	20,8
Potencialmente evitável	72	79,1	47	62,7	29	58	148	68,5
Potencialmente não evitável	9	9,9	8	10,7	4	8	21	9,7
Fortemente não evitável	0	0	0	0	2	4	2	0,1

A classificação dos tipos de EA ocorridos antes e após as intervenções está apresentada na Tabela 4. Observou-se, entre os períodos pré-intervenção e pós-intervenções I e II, uma redução significativa na prevalência de pacientes acometidos por retenção urinária (33%; 14,4%; 3,1%, p< 0,001) e por hemorragia (9,3%; 7,2%; 0%, p = 0,012).

O oposto ocorreu para casos de lesões de pele (lesão por posicionamento cirúrgico/lesão por dispositivo/adesivo médico), que aumentaram de 2,1% (pré-intervenção) para 10,3% (pós-intervenções) (p = 0,043). Não foi identificada diferença significativa quanto à prevalência de pacientes com infecção de ferida cirúrgica (6,2%; 4,1%; 5,2%, p = 0,810) e hematoma/seroma (5,2%; 9,3%; 8,3%, p = 0,528).

**Tabela 4 -:** Distribuição dos eventos adversos antes e após a implementação de *checklists*cirúrgicos segundo o tipo. Curitiba, PR, Brasil, 2022

**Tipo de evento adverso**	**Período de intervenção**						**Total (n = 216)**	
	Pré-intervenção (n = 91)		Pós-intervenção I (n = 75)		Pós-intervenção II (n = 50)			
	n	%	n	%	n	%	n	%
Retenção urinária	32	35,2	14	%	3	6	49	22,7
Hematoma/seroma	5	5,5	9	12	8	16	22	10,2
Hemorragia	9	9,9	7	9,3	0	0	16	7,4
Deslocamento de cateter peridural	7	7,7	7	9,3	1	2	15	6,9
Infecção de ferida	6	6,6	4	5,3	5	10	15	6,9
Lesão de mucosa/pele	2	2,2	7	9,3	6	12	15	6,9
Náuseas/vômitos	6	6,6	3	4	2	4	11	5,1
Lesão por posicionamento cirúrgico	0	0	4	5,3	5	10	9	4,2
Trombose venosa profunda	3	3,3	2	2,7	2	4	7	3,2
Falta de material/equipamento/leito	2	2,2	2	2,7	1	2	5	2,3
Dor aguda	0	0	4	5,3	0	0	4	1,9
Fratura	3	3,3	1	1,3	0	0	4	1,9
Luxação	1	1,1	0	0	3	6	4	1,9
Neuropraxia	1	1,1	3	4	0	0	4	1,9
Prurido	0	0	3	4	1	2	4	1,9
Deiscência de sutura	1	1,1	1	1,3	1	2	3	1,4
Dismetria de membro inferior	2	2,2	1	1,3	0	0	3	1,4
Falha de bloqueio anestésico	1	1,1	0	0	2	4	3	1,4
Falha de equipamento	1	1,1	0	0	2	4	3	1,4
Soltura de material de síntese	1	1,1	1	1,3	1	2	3	1,4
Tromboembolismo pulmonar	0	0	0	0	3	6	3	1,4
Hipotensão	2	2,2	0	0	0	0	2	0,9
Perfuração de dura-máter	1	1,1	1	1,3	0	0	2	0,9
Reação transfusional	0	0	0	0	2	2	2	0,9
Acidente vascular cerebral	1	1,1	0	0	0	0	1	0,5
Choque séptico	1	1,1	0	0	0	0	1	0,5
Enfisema subcutâneo	1	1,1	0	0	0	0	1	0,5
Erro de medicação	1	1,1	0	0	0	0	1	0,5
Fístula	0	0	0	0	1	2	1	0,5
Hipoglicemia	1	1,1	0	0	0	0	1	0,5
Hipóxia	0	0	0	0	1	2	1	0,5
Intoxicação por opioide	0	0	1	1,3	0	0	1	0,5

Quanto à frequência de readmissões decorrentes do EA, observou-se que não houve diferença significativa antes e após a implementação dos *checklists* (p = 0,092): pré-intervenção (9,7%; n = 6), pós-intervenção I (8,2%; n = 4) e pós-intervenção II (22,9%; n = 8).

## Discussão

A redução da prevalência de EA entre pacientes submetidos à artroplastia de quadril e joelho requer a compreensão dos distintos fatores que colaboram para o aumento dos riscos assistenciais e cirúrgicos, bem como o correspondente julgamento crítico dos gestores quanto às intervenções previstas no planejamento estratégico institucional para promover práticas seguras e proteger os pacientes de erros e danos cirúrgicos. Nesta pesquisa, constatou-se um declínio significativo da prevalência e do número de EA por paciente após a aplicação dos dois *checklists* cirúrgicos. Esses resultados somam-se às evidências que indicam que o uso de listas de verificação de segurança cirúrgica, ou *checklists*, tem proporcionado práticas seguras e baseadas em valor^(28)^.

O resultado favorável apresentado na presente pesquisa é potencialmente fruto do empenho dos profissionais de saúde em adaptar, elaborar e implantar, gradativamente, *checklists* cirúrgicos para atender às recomendações do PCSSV, bem como da participação das lideranças na construção progressiva de uma cultura de segurança do paciente, fator que contribui para a visibilidade dos riscos e a adoção de ações preventivas.

Resultados satisfatórios a partir da implementação do *checklist* cirúrgico também foram observados em um estudo conduzido em um hospital terciário da Austrália, cujos autores apontaram uma redução significativa na taxa de mortalidade de 1,2% para 0,92%^(29)^. Da mesma forma, uma investigação realizada em hospitais de ensino do Reino Unido mostrou uma diminuição na prevalência de EA, de 16,9% para 11,2% (p = 0,01)^(30)^.

Na Noruega, pesquisadores apontaram que o uso concomitante de uma lista de verificação aplicada no pré e pós-operatório, juntamente com o *checklist* utilizado no intraoperatório, reduziu significativamente os EA, reoperações e readmissões não planejadas^(31)^. Esse dado diverge, em parte, dos resultados da presente pesquisa, em que não houve diferença nas readmissões decorrentes desses agravos. Vale destacar que os efeitos positivos dos *checklists* também são demonstrados em serviços cirúrgicos de países de baixa e média renda, como é o caso do Brasil, com uma redução de 44% na incidência geral de EA^(32)^, corroborando os achados aqui apresentados.

Nesta pesquisa, a redução significativa de pacientes com retenção urinária após as intervenções pode ser explicada, principalmente, pelo decréscimo observado do uso de anestesia peridural e analgesia aguda por cateter peridural na fase pós-operatória. Outro fator potencialmente relacionado é a adesão às diretrizes baseadas em evidências para prevenir o agravo, com o uso cauteloso de opioides administrados por via intratecal e a associação de distintas drogas analgésicas para o tratamento da dor aguda^(33-34)^, inclusive desde o período pré-operatório, visando reduzir o consumo de opiáceos no pós-operatório^(35)^.

Reitera-se que a adoção do *checklist* possibilita melhorar o planejamento pré-operatório e antecipar eventos críticos mediante a identificação de fatores intrínsecos e extrínsecos que aumentam o risco clínico-cirúrgico de episódios de retenção urinária^(36)^. Esse achado é relevante, pois tal EA potencializa problemas relacionados ao músculo vesical, comprometendo sua funcionalidade e acentuando o risco de infecções urinárias, com consequente necessidade de procedimentos complementares e sucessivo aumento dos riscos e do tempo de internação hospitalar^(36-37)^.

Quanto aos impactos do *checklist* na prevenção de hemorragias, observam-se divergências na literatura ao considerar os distintos cenários perioperatórios. Enquanto um estudo conduzido na Noruega revelou diminuição dos casos de sangramento pós-operatório de 2,6% para 1,0% (p< 0,001), bem como dos custos associados à transfusão de hemocomponentes^(38)^, outra investigação realizada em um centro de cuidados terciários na Austrália constatou não haver diferença na ocorrência desse EA^(29)^.

A diferença significativa encontrada nesta pesquisa quanto ao número de pacientes expostos a esse EA, antes e após as intervenções, pode estar relacionada a melhorias da avaliação do paciente, no uso de equipamentos e nas técnicas operatórias empregadas. Reconhece-se que o PCSSV, na etapa pré-operatória imediata, busca antecipar a possibilidade de perda sanguínea e permitir o planejamento de ações multimodais, como a reserva prévia de fatores lábeis de coagulação e hemocomponentes, com o objetivo de minimizar e tratar essa eventualidade, além de evitar EA graves^(39)^. Assim, considerando tratar-se de cirurgias eletivas, a avaliação realizada pelas equipes cirúrgica, de anestesiologia e de enfermagem no pré-operatório oferece uma oportunidade para a prevenção do dano.

Há também que se considerar que se trata de um hospital de ensino e, como tal, a baixa experiência de residentes na realização de técnicas operatórias representa um fator de risco para o paciente e um desafio para os coordenadores e profissionais atuantes nos núcleos de segurança do paciente. A supervisão direta dos aprendizes colabora para uma formação de qualidade e um aprendizado seguro, promovendo a segurança do paciente por meio da otimização do tempo operatório das artroplastias de quadril e joelho. Além disso, melhorias relacionadas à gestão do cuidado de enfermagem no pós-operatório, como a avaliação e manejo de curativos e drenos cirúrgicos, e a aplicação de crioterapia podem ter contribuído para a redução dos eventos hemorrágicos^(40)^. O *checklist* institucional, aplicado no período pós-operatório imediato, inclui a verificação de sinais de sangramento, cuja detecção precoce contribui para evitar o agravamento do caso^(19)^.

A implementação das duas modalidades de *checklist* não interferiu na redução do tempo de internação hospitalar, contrariando a literatura^(29)^. Infere-se que a maior gravidade e complexidade dos pacientes possam ter contribuído para a manutenção do tempo do processo anestésico-cirúrgico e da permanência no hospital. Outrossim, constatou-se um aumento da prevalência de pacientes com danos ao tecido cutâneo, caracterizados como lesões por posicionamento cirúrgico e/ou lesões causadas por dispositivo ou adesivo médico. Distintos fatores podem ter colaborado para a ocorrência desses eventos na amostra estudada, os quais podem ser explorados em pesquisas subsequentes. Porém, a redução do tempo operatório^(41)^ e o uso de dispositivos e superfícies de suporte adequados^(42-43)^ são fatores protetores contra esses EA e que merecem ser abordados gerencialmente, à luz da formação e da prática profissional, com destaque à equipe de enfermagem.

Por outro lado, destaca-se que, antes das intervenções, esses agravos poderiam ser percebidos pela equipe de saúde como ocorrências comuns na área cirúrgica, recebendo, consequentemente, menor importância quanto ao registro e notificação em comparação aos demais EA. Com a ascensão dos aspectos relativos ao gerenciamento de riscos e o aprimoramento de protocolos para a prevenção de incidentes cirúrgicos, houve um potencial estímulo ao amadurecimento da cultura de segurança do paciente ao longo do período da pesquisa. Esse avanço pode ter contribuído para melhorias nos processos de trabalho, elevando a percepção e o compromisso dos profissionais com a identificação e o registro desses EA. Como consequência, tornou-se possível detectar, no rastreio documental, eventos anteriormente não registrados nos prontuários dos pacientes.

Não foi identificada uma diminuição significativa na prevalência de pacientes com infecção de ferida cirúrgica e hematomas/seromas, diferentemente do que foi constatado em hospitais da Etiópia, onde a prevalência de ISC passou de 27,3% para 9,1% (p= 0,086)^(44)^, e do que foi relatado por pesquisadores da Índia, que observaram uma redução significativa desse tipo de EA antes e após a implementação do *checklist* (14% para 9,5%)^(45)^. Contudo, é reconhecido que o uso dessas ferramentas, de forma isolada, tem efeito limitado como medida de prevenção de ISC^(46)^, pois sua ocorrência é de natureza multifatorial, envolvendo fatores relacionados ao ambiente, ao paciente e ao procedimento cirúrgico^(47)^.

Não obstante, observa-se que a prevalência de EA permanece elevada no hospital do estudo, sendo a maioria desses eventos evitáveis, conforme estudo de coorte multicêntrico envolvendo 24 hospitais da Suécia, que apontou prevalência de EA em artroplastias de quadril de 58,7%, com 76% dos registros classificados como preveníveis^(48)^. No Brasil, os estudos epidemiológicos para avaliação da gravidade e evitabilidade desses agravos, exclusivamente na área ortopédica, são incipientes. Contudo, observa-se que a incidência de EA de 33,7% foi evidenciada em um hospital de ensino situado na região sudeste brasileira, com mais da metade dos casos sendo evitáveis^(49)^.

Os resultados apresentados nesta pesquisa contribuem para evidenciar a potencial contribuição da implementação de *checklists* na redução de EA e reiteraram a relevância da adoção de boas práticas assistenciais e seu monitoramento, por meio de estudos epidemiológicos. Outro aspecto contributivo desta pesquisa ao conhecimento científico está relacionado à metodologia de rastreamento de EA adotada, que é replicável em diferentes culturas e contextos assistenciais. A identificação ativa de EA contribui para reconhecer e classificar desfechos desfavoráveis, para a construção de indicadores de qualidade, e para subsidiar o planejamento e a revisão de protocolos assistenciais.

Ademais, os resultados contribuem para estimular outros pesquisadores a investigarem o tema em cenários distintos, pois a utilização dessas ferramentas está fortemente influenciada pelas particularidades de cada unidade assistencial, considerando aspectos tecnológicos, demográficos, epidemiológicos e os modelos de gestão das organizações de saúde, além da adesão da equipe multidisciplinar aos princípios de qualidade.

Entre as limitações da presente pesquisa, destacam-se a análise retrospectiva de base documental, que depende da qualidade dos registros, o tamanho amostral e a realização do estudo em um único hospital. Além disso, reconhece-se que múltiplos fatores podem interferir na qualidade assistencial e na ocorrência de EA, não se restringindo às intervenções aqui estudadas.

## Conclusão

Houve redução na frequência e prevalência geral de EA após a implementação de *checklists* cirúrgicos, especialmente na diminuição de retenção urinária e hemorragia. Contudo, constatou-se aumento na prevalência de lesões de pele. Não foram observados efeitos relativos à ocorrência de infecção de ferida operatória e hematomas/seromas, assim como no tempo de internação hospitalar e nas readmissões hospitalares decorrentes de EA.

# Translation: Prevalencia de eventos adversos en artroplastias de cadera y rodilla tras la aplicación de listas de verificación quirúrgicas*

**DOI:** 10.1590/1518-8345.7658.4556

**Authors:** Josemar Batista, Elaine Drehmer de Almeida Cruz

**Affiliations:** 1 Universidade Federal do Paraná, Departamento de Enfermagem, Curitiba, PR, Brasil; 2 Universidade Positivo, Escola de Ciências da Saúde, Curitiba, PR, Brasil

**Keywords:** Atención Perioperativa, Errores Médicos, Seguridad del Paciente, Artroplastia, Lista de Verificación, Evaluación de Procesos y Resultados en Atención de Salud

## Abstract

identificar la prevalencia de eventos adversos en pacientes sometidos a artroplastias de cadera y rodilla tras la implementación de listas de verificación quirúrgicas.

investigación evaluativa del tipo análisis de efectos, realizada en tres períodos: antes (0- 2010) y después de la intervención (I- 2013; II- 2016), mediante consulta retrospectiva en una muestra aleatoria simple de 291 historias clínicas, entre noviembre de 2020 y marzo de 2022. Se utilizaron los formularios del *Canadian Adverse Events Study* y *Global Trigger Tool* para rastrear y confirmar eventos adversos. Los casos fueron analizados mediante estadística descriptiva e inferencial; valores p ≤ 0,05 indicaron significancia estadística.

en los períodos posteriores a la implementación de listas de verificación quirúrgicas, se observó una reducción en la frecuencia de pacientes afectados por dos o más eventos, del 27,8 % al 11,3 % (p = 0,002), y en la prevalencia general, del 63,9 % al 36,1 % (p < 0,001). Hubo una disminución en la prevalencia de pacientes afectados por retención urinaria (33 % al 3,1 %; p < 0,001) y hemorragia (9,3 % al 0 %; p = 0,012). Se registró un aumento en la prevalencia de lesiones cutáneas, del 2,1 % al 10,3 % (p = 0,043).

hubo una reducción en la prevalencia general y en la frecuencia de eventos adversos en pacientes sometidos a artroplastia tras la implementación de listas de verificación.

## Destacados

**(1)** Alta prevalencia de eventos adversos en artroplastias de cadera y rodilla. **(2)** Listas de verificación quirúrgicas reducen la prevalencia de eventos adversos en artroplastias. **(3)** Hubo una disminución significativa de pacientes afectados por dos o más eventos adversos. **(4)** Investigar el impacto de las intervenciones contribuye a promover una cultura de seguridad. **(5)** Los resultados son relevantes para fortalecer acciones básicas de seguridad perioperatoria.

## Introducción

Las evidencias de que la mortalidad quirúrgica es superior entre quienes reciben cuidados de menor calidad, en comparación con aquellos que no tienen acceso a los servicios quirúrgicos, reiteran la importancia de acciones innovadoras dirigidas a la oferta de mejores prácticas en el contexto perioperatorio^(1-2)^. Este dato refuerza el nivel de criticidad del sector, tanto en las exigencias orientadas a las tareas y procesos como en las demandas cognitivas de los profesionales de la salud, con el objetivo de ofrecer cuidados de excelencia^(2)^.

Se reconoce que la complejidad del área quirúrgica contribuye a la ocurrencia de errores e incidentes relacionados con la asistencia, especialmente en intervenciones que requieren el uso de herramientas e implantes, como es el caso de las artroplastias de rodilla y cadera^(3-5)^. Conceptualmente, el error consiste en la falla al ejecutar una acción planificada según lo previsto o en aplicar un plan de forma incorrecta. Estas situaciones aumentan el riesgo de incidentes con daños al paciente, definidos como eventos adversos (EA)^(6-7)^.

En la atención ortopédica, independientemente del tipo de anestesia y del procedimiento quirúrgico al que se someta el paciente, los errores no son poco frecuentes. Existe consenso en que esta especialidad quirúrgica presenta una mayor probabilidad de daños evitables en comparación con otras^(3,8-9)^. Hay un mayor riesgo de cirugías en el lugar equivocado^(10)^ y de eventos infecciosos graves que requieren reintervención quirúrgica^(11-12)^, principalmente debido a las particularidades inherentes a la implantación de materiales^(13)^. La falta de estandarización en equipos, procedimientos y protocolos en esta especialidad quirúrgica se suma a los riesgos y potencia la ocurrencia de daños^(14)^.

Ante los distintos factores críticos que contribuyen a errores y eventos adversos quirúrgicos en general, la Organización Mundial de la Salud lanzó, en el bienio 2007-2008, el programa La Cirugía Segura Salva Vidas (PCSSV), recomendando el uso de listas de verificación quirúrgicas y su adaptación opcional para atender demandas locales y regionales. El instrumento, en su versión original, contiene 19 ítems y tiene como objetivo garantizar la seguridad del paciente^(15-16)^. Desde entonces, investigaciones evidencian los beneficios de esta herramienta, impactando en una reducción del 33% de eventos adversos^(16-17)^, con resultados que refuerzan su importancia en la promoción de la calidad asistencial y la prevención de errores.

En ese contexto, las acciones de seguridad destinadas a cumplir los objetivos del referido programa se adoptaron de forma gradual en el hospital de esta investigación. En 2011, la lista de verificación quirúrgica recomendada por la OMS fue adaptada y utilizada de manera pionera por la especialidad de ortopedia en cirugías con implantes^(18)^. En el segundo semestre de 2014, se implementó otra lista de verificación para su aplicación en la unidad de hospitalización ortopédica, enfocada en la verificación de ítems de seguridad relacionados con las fases pre y postoperatorias^(19)^.

Al analizar los recursos humanos y financieros invertidos en el área quirúrgica, así como las adaptaciones y/o la elaboración de ítems de verificación para la consolidación de acciones y políticas institucionales en el hospital objeto de estudio, se consideró importante llevar a cabo estudios evaluativos. Estos tienen como finalidad evaluar los efectos de la implementación de dichas herramientas en el contexto asistencial, mediante el análisis antes y después de la intervención.

Así, el objetivo de esta investigación fue identificar la prevalencia de eventos adversos en pacientes sometidos a artroplastias de cadera y rodilla tras la implementación de listas de verificación quirúrgicas.

## Método

### Diseño del estudio

Se trata de una investigación evaluativa del tipo análisis de efectos^(20)^, documental, retrospectiva y con enfoque cuantitativo.

### Escenario del estudio

La investigación se llevó a cabo en un hospital universitario de gran porte en la región sur de Brasil, que atiende exclusivamente a través del *Sistema Único de Saúde*. La especialidad quirúrgica involucrada fue ortopedia, y se consideraron los datos de internaciones de pacientes sometidos a artroplastias de cadera y rodilla en distintos períodos, correspondientes a antes y después de la implementación de dos modalidades de listas de verificación quirúrgicas. Estos períodos se denominan aquí pre-intervención y post-intervenciones I y II. La elección de esta población se justifica específicamente por la complejidad quirúrgica del procedimiento con implante de prótesis y por el carácter pionero de la especialidad en el uso de estos instrumentos.

La intervención I ocurrió en 2011 con la implementación de una lista de verificación para uso en el centro quirúrgico, compuesta por 45 ítems organizados en cuatro momentos: (I) recepción del paciente, (II) antes de la inducción anestésica, (III) antes de la incisión quirúrgica, y (IV) antes de que el paciente abandone la sala de operaciones^(18)^. En 2014, se implementó una lista de verificación para uso en la unidad de internación ortopédica (Intervención II), con 97 ítems de seguridad distribuidos en seis momentos: (1) Identificación; (2) Preoperatorio: ítems a verificar antes de que el paciente sea trasladado al centro quirúrgico; (3) Postoperatorio inmediato: verificación de indicadores de seguridad durante las primeras 24 horas después del procedimiento anestésico-quirúrgico; (4) Postoperatorio mediato: ítems relacionados con la evaluación del dolor, los aspectos de la herida quirúrgica y la restauración de los sistemas fisiológicos; (5) Complicaciones: registro de complicaciones postoperatorias, como infecciones; y (6) Alta hospitalaria/transferencia: ítems de seguridad relacionados con el estado general del paciente, las condiciones del sitio quirúrgico y de los dispositivos, además de orientaciones para cuidados domiciliarios y seguimiento ambulatorio^(19)^.

Ambas listas de verificación incluyen la comprobación de elementos que contribuyen a la prevención de errores y la detección de no conformidades relacionadas con la seguridad y la calidad asistencial. Se adoptó un intervalo de dos años para la investigación y el análisis, correspondiente al año 2010 (pre-intervención) y a los años 2013 y 2016 (post-intervención I y II).

### Población y criterios de selección

La fuente de información de interés para el estudio fue la base de datos proporcionada por el servicio de informática del hospital, compuesta por la totalidad de las cirugías de artroplastias de cadera y rodilla realizadas en el período del 1 de enero al 31 de diciembre de 2010 (pre-intervención), del 1 de enero al 31 de diciembre de 2013 (post-intervención I) y del 1 de enero al 31 de diciembre de 2016 (post-intervención II). Se eliminaron los registros de historias clínicas repetidos, manteniéndose únicamente aquellos relativos a la primera cirugía realizada durante la hospitalización analizada, correspondiente a la hospitalización índice.

Se incluyeron las historias clínicas de pacientes adultos con un tiempo mínimo de hospitalización de 24 horas. También se incluyeron los casos de fallecimientos ocurridos durante el intraoperatorio y con un período de hospitalización inferior a 24 horas. Se excluyeron los casos con diagnóstico relacionado con enfermedades psiquiátricas, según lo previamente establecido^(21-22)^.

### Definición de la muestra

Para estimar el tamaño muestral, se realizó un estudio piloto para detectar diferencias significativas en la prevalencia de EA al comparar los períodos de pre-intervención y post-intervención II. La estimación inicial de la prevalencia se obtuvo a partir de una muestra piloto de 25 historias clínicas seleccionadas aleatoriamente para cada condición, en la que se obtuvo una prevalencia del 60% y 40% de EA, respectivamente, aplicando la metodología de recolección de datos que se describe a continuación. Para el cálculo de la muestra, se consideró un nivel de significancia del 5% y un poder estadístico del 80%, lo que resultó en un tamaño mínimo de muestra de 97 historias clínicas para cada condición.

La selección aleatoria simple de las historias clínicas elegibles se realizó a partir de una lista general de cirugías generada por la base de datos institucional, utilizando el *software Microsoft Office Excel* 2016^®^. Las historias clínicas no disponibles en el servicio de archivo físico fueron reemplazadas por las historias clínicas inmediatamente siguientes en dicha lista, sin pérdidas insustituibles. Las historias clínicas utilizadas en el estudio piloto fueron incluidas en la investigación.

### Recolección de datos e instrumentos utilizados

La recolección de datos se realizó entre los meses de noviembre de 2020 y marzo de 2022, mediante consulta retrospectiva exclusivamente de la información contenida en las historias clínicas físicas, y sistematizada en dos fases, conforme a la metodología adaptada del protocolo del *Canadian Adverse Events Study* ^(21)^. La Fase I (revisión primaria) fue conducida por un único enfermero e investigador principal, consistiendo en la búsqueda e identificación de potenciales eventos adversos (pEA), guiada por dos formularios de rastreo.

El primer formulario corresponde al protocolo del *Canadian Adverse Events Study*, compuesto por 17 criterios explícitos de rastreo de pEA relacionados con la cirugía, anestesia, medicamentos, diagnóstico, cuidados y tratamientos no medicamentosos^(21)^. Este protocolo fue traducido y adaptado para su uso en Brasil^(22)^. El segundo formulario de rastreo se refiere al módulo quirúrgico del *Global Trigger Tool*, desarrollado por el *Institute for Healthcare Improvement* de los Estados Unidos de América. Se aplicó con el objetivo de ampliar la búsqueda de rastreadores de EA, y está compuesto por 11 desencadenantes para la identificación de pEA ocurridos en los períodos intra y postoperatorios^(23)^, traducidos y adaptados transculturalmente para el contexto brasileño^(24)^.

Para la identificación de pEA ocurridos después del alta hospitalaria, se utilizaron los registros contenidos en las fichas de consultas ambulatorias que forman parte de los prontuarios de los pacientes. En los casos de Infección de Sitio Quirúrgico (ISQ), se adoptaron los criterios de los *Centers for Disease Control and Prevention* que definen la infección como aquella que ocurre hasta 30 días y/o 90 días después de un procedimiento quirúrgico con inserción de implante^(25)^.

En los casos en los que se identificó al menos un criterio de rastreo, se completó el formulario de evaluación de pEA^(21-22)^ y el guion semiestructurado para la caracterización del perfil demográfico, clínico, quirúrgico y anestésico, elaborado para la investigación.

Los registros de la Fase I (revisión primaria) fueron analizados en la Fase II (revisión secundaria), correspondiente a la etapa de confirmación o descarte del EA, mediante consenso de un comité de expertos^(21-22)^. Este comité fue formado, de manera intencional, por el investigador principal, un enfermero y un médico, todos con título de maestría y con más de 10 años de experiencia en el área de gestión de calidad y seguridad del paciente.

Para juzgar los pEA, se utilizaron los formularios completados en la etapa anterior. Para confirmar o descartar el caso, se adoptó el concepto de EA propuesto por la OMS^(6-7)^, y se utilizaron tres escalas. La primera evaluó la gravedad del EA^(6)^. La segunda y la tercera escalas se aplicaron para determinar, respectivamente, si el daño fue causado por la atención prestada al paciente y el grado de evitabilidad del evento. Estas escalas están compuestas por seis puntuaciones, que van desde (1) “prácticamente ninguna evidencia” hasta (6) “evidencia casi cierta”. Se consideraron EA evitables aquellos con puntuaciones ≥ 4 puntos^(21,26)^.

### Variables del estudio

Las variables cuantitativas y/o categóricas registradas fueron: sexo, edad (en años), clasificación de la cirugía (electiva; emergencia), potencial de contaminación (limpia; potencialmente contaminada; contaminada; infectada), tipo de anestesia (inhalatoria/sedación; general; raquídea; epidural; bloqueo; local), y riesgo quirúrgico según la clasificación de la *American Society of Anesthesiologists* – ASA. Además, se consideraron las comorbilidades (factores de riesgo intrínsecos) informadas en la ficha de evaluación pre-anestésica y el uso de dispositivos médico-hospitalarios (factores de riesgo extrínsecos). También se extrajeron datos para medir el tiempo de hospitalización del paciente en el preoperatorio (< 24 horas; ≥ 24 horas) y el tiempo de permanencia hospitalaria (en días).

Los EA fueron clasificados en: (1) leve; (2) moderado; (3) grave; y (4) óbito^(6)^. El grado de evitabilidad de los EA se definió con la siguiente subclasificación: (A) Fuertemente evitable (puntuación 6); (B) Potencialmente evitable (puntuaciones 4 y 5); (C) Potencialmente no evitable (puntuaciones 2 y 3); y (D) Fuertemente no evitable (puntuación 1)^(27)^.

Los EA confirmados también fueron categorizados según la Clasificación Internacional para la Seguridad del Paciente en la clase 1 (tipo de incidente), distribuidos en las siguientes categorías: (A) Administración clínica; (B) Proceso/procedimiento clínico; (C) Documentación; (D) Infección relacionada con la asistencia sanitaria (IRAS); (E) Medicación/fluidos intravenosos; (F) Sangre/hemoderivados; (G) Nutrición; (H) Oxígeno/gases/vapores; (I) Dispositivos/equipos médicos; (J) Comportamiento; (K) Accidentes con el paciente; (L) Infraestructura/lugar/instalaciones; y (M) Recursos/gestión organizacional^(6)^.

### Procesamiento y análisis de los datos

Los datos recolectados fueron ingresados, mediante doble digitación, en una hoja de cálculo de *Microsoft Office Excel* 2016^®^. Después de la verificación y corrección de inconsistencias, se analizaron con asesoría estadística utilizando el *software International Business Machines Corporation™ - Statistical Package for the Social Sciences* (IBM™ - SPSS) versión 20.0 para *Windows.*

Las medidas utilizadas fueron: prevalencia de EA entre los pacientes: [(número de pacientes con al menos un EA/número total de pacientes) x 100]; y proporción de EA evitables [(número de EA evitables/número total de EA) x 100].

Las variables cuantitativas fueron descritas mediante estadística descriptiva univariada, mientras que las categóricas se presentaron por frecuencias absolutas y relativas. Para comparar los períodos pre-intervención, intervención I e intervención II en las variables cuantitativas, se utilizó el modelo de análisis de varianza (ANOVA) con un factor o la prueba no paramétrica de Kruskal-Wallis. Para comparaciones múltiples, se aplicó la prueba *post-hoc* de *Dunn*, con los valores de p corregidos por el método de Bonferroni. La normalidad de las variables cuantitativas se evaluó mediante la prueba de Kolmogorov-Smirnov. Valores de p ≤ 0,05 indicaron significancia estadística.

### Aspectos éticos

La investigación fue aprobada por el comité de ética institucional bajo el parecer número 3.651.686. Se solicitó la exención de la obtención del Formulario de Consentimiento Informado (FCI) relacionado con los pacientes. Los revisores/especialistas invitados a participar en la Fase II formalizaron su aceptación mediante la firma del FCI.

## Resultados

En la revisión primaria de los 291 expedientes, se constató que la edad promedio (respectivamente en las fases pre-intervención, post-intervención I y post-intervención II) fue de 57,2 años (desviación estándar ‒ DE ± 14,4), 56,5 (DE ± 15,5) y 59,5 (DE ± 15,4), sin diferencia significativa entre los períodos analizados (p = 0,355). En cuanto a los factores de riesgo intrínsecos de los pacientes, se observó que no hubo diferencia entre pre y post-intervenciones (p = 0,822). Las demás características demográficas y clínicas están presentadas en la Tabla 1.

**Tabla 1 -:** Perfil demográfico-clínico de los pacientes sometidos a artroplastias de cadera y rodilla antes y después de la implementación de listas de verificación quirúrgicas. Curitiba, PR, Brasil, 2022

**Variable**	**Pre-intervención (n=97)**	**Post-intervención I (n=97)**	**Post-intervención II (n=97)**	**p-valor** *
	**n (%)**	**n (%)**	**n (%)**	
**Sexo**				
Femenino	56 (57,7)	59 (60,8)	62 (63,9)	0,677
Masculino	41 (42,3)	38 (39,2)	35 (36,1)	
**Comorbilidad/factor de riesgo**				
Hipertensión arterial sistémica				
Sí	58 (59,8)	50 (51,6)	56 (57,7)	0,484
No	39 (40,2)	47 (48,5)	41 (42,3)	
Tabaquismo				
Sí	15 (15,5)	25 (25,8)	13 (13,4)	0,057
No	82 (84,5)	72 (74,2)	84 (86,6)	
Diabetes *mellitus*				
Sí	12 (12,4)	14 (14,4)	19 (19,6)	0,359
No	85 (87,6)	83 (85,6)	78 (80,4)	
Neumopatía ^†^				
Sí	10 (10,3)	6 (6,2)	6 (6,2)	0,455
No	87 (89,7)	91 (93,8)	91 (93,8)	
Enfermedades de la tiroides ^‡^				
Sí	9 (9,3)	11 (11,3)	7 (7,2)	0,613
No	88 (90,7)	86 (88,7)	90 (92,8)	
Artritis reumatoide				
Sí	6 (6,2)	3 (3,1)	3 (3,1)	0,457
No	91 (93,8)	94 (96,9)	94 (96,9)	
Cardiopatía				
Sí	6 (6,2)	7 (7,2)	7 (7,2)	0,948
No	91 (93,8)	90 (92,8)	90 (92,8)	
Hepatitis				
Sí	5 (5,2)	7 (7,2)	4 (4,1)	0,629
No	92 (94,9)	90 (92,8)	93 (95,9)	
Dislipidemia				
Sí	4 (4,1)	2 (2,1)	5 (5,2)	0,516
No	93 (95,9)	95 (97,9)	92 (94,9)	
Alcoholismo				
Sí	4 (4,1)	4 (4,1)	6 (6,2)	0,741
No	93 (95,9)	93 (95,9)	91 (93,8)	
Hemofilia				
Sí	3 (3,1)	10 (10,3)	8 (8,3)	0,135
No	94 (96,9)	87 (89,7)	89 (91,8)	
Neoplasia				
Sí	2 (2,1)	2 (2,1)	1 (1)	-
No	95 (97,9)	95 (97,9)	96 (99)	
Osteoporosis				
Sí	2 (2,1)	2 (2,1)	0 (0)	-
No	95 (97,9)	95 (97,9)	97 (100)	

*Prueba de Chi-cuadrado, p< 0,05; ^†^Neumopatía = Asma, bronquitis, edema pulmonar agudo, enfisema pulmonar, enfermedad pulmonar obstructiva crónica; ^‡^Enfermedades de la tiroides = Hipotiroidismo, hipertiroidismo

El tiempo promedio de hospitalización fue de 3,7 días (DE ± 1,8) en 2010 (pre-intervención), 4,9 días (DE ± 3,7) en 2013 (post-intervención I) y 4,6 días (DE ± 4,4) en 2016 (post-intervención II), con una diferencia significativa entre los períodos (p = 0,008), destacándose entre 2010 y 2013 (p = 0,006).

Se observó, durante la hospitalización en todos los períodos analizados, la presencia de factores de riesgo extrínsecos, destacándose el uso de catéter venoso periférico (100% en todos los períodos) y catéter epidural (88,7%; 71,1% y 4,1%, respectivamente). Este último mostró una reducción significativa (p < 0,001) en su uso antes y después de las intervenciones I y II, respectivamente. Hubo una reducción significativa de pacientes sometidos a anestesia epidural (p < 0,001) y sedación (p = 0,008); ocurrió lo contrario para la anestesia general (p = 0,001), tal como se muestra en la Tabla 2, junto con otras características de interés.

**Tabla 2 -:** Distribución del perfil quirúrgico-anestésico y de hospitalización de pacientes sometidos a artroplastias de cadera y rodilla antes y después de la implementación de listas de verificación quirúrgicas. Curitiba, PR, Brasil, 2022

**Variable**	**Pre-intervención (n=97)**	**Post-intervención I (n=97)**	**Post-intervención II (n=97)**	**p-valor** *
	**n (%)**	**n (%)**	**n (%)**	
**Diagnóstico preoperatorio**				-
Gonartrosis	36 (37,1)	37 (38,1)	46 (47,4)	
Coxartrosis	32 (33)	34 (35,1)	36 (37,1)	
Aflojamiento aséptico de componente	12 (12,4)	8 (8,3)	8 (8,3)	
Coxartrosis secundaria	7 (7,2)	3 (3,1)	2 (2,1)	
Gonartrosis secundaria	4 (4,1)	11 (11,3)	3 (3,1)	
Otro ^†^	6 (6,2)	4 (4,1)	2 (2)	
**Tiempo de hospitalización preoperatoria** 0,343				
< 24 horas	91 (93,8)	88 (90,7)	93 (95,9)	
≥ 24 horas	6 (6,2)	9 (9,3)	4 (4,1)	
**Procedimiento quirúrgico**				0,220
Artroplastia total de cadera	44 (45,4)	43 (44,3)	36 (37,1)	
Artroplastia total de rodilla	37 (38,1)	45 (46,4)	51 (52,6)	
Artroplastia de revisión de cadera	9 (9,3)	8 (8,3)	6 (6,2)	
Artroplastia de revisión de rodilla	7 (7,2)	1 (1)	4 (4,1)	
**Clasificación de la cirugía**				-
Electiva	95 (97,9)	97 (100)	97 (100)	
Emergencia	2 (2,1)	0 (0)	0 (0)	
**Potencial de contaminación**				-
Limpia	97 (100)	97 (100)	96 (99)	
Infectada	0 (0)	0 (0)	1 (1)	
**Riesgo quirúrgico ASA** ^‡^				0,271
I	22 (22,7)	14 (14,4)	12 (12,4)	
II	61 (62,9)	72 (74,2)	70 (72,2)	
III	14 (14,4)	11 (11,3)	15 (15,5)	
**Tipo de anestesia** ^§^				
Epidural	86 (88,7)	65 (67)	7 (7,2)	< 0,001
Raquídea	73 (75,3)	74 (76,3)	70 (72,2)	0,790
Sedación	71 (73,2)	61 (62,9)	50 (51,6)	0,008
General	21 (21,7)	23 (23,7)	43 (44,3)	0,001
Bloqueo	0 (0)	2 (2,1)	0 (0)	-
Local	0 (0)	1 (1)	4 (4,1)	-

*Prueba de Chi-cuadrado, p< 0,05; ^†^Otro = Fractura del cuello femoral, rotura del material de síntesis, osteonecrosis del fémur, fractura periprotésica, fractura de fémur, artropatía hemofílica, infección operatoria; ^‡^ASA = *American Society of Anesthesiologists*; ^§^Un mismo paciente puede ser sometido a más de un tipo de anestesia

La prevalencia de EA fue del 63,9%, 50,5% y 36,1%, respectivamente, para los períodos pre-intervención, post-intervención I y post-intervención II, con una reducción significativa (p < 0,001). La detección del EA ocurrió predominantemente durante la hospitalización, con una diferencia significativa entre los períodos pre y post-intervenciones (p = 0,046).

En cuanto al número de EA por paciente, se observó un promedio de 1,47 EA por paciente en el período pre-intervención, 1,53 EA por paciente en el post-intervención I y 1,43 por paciente en el post-intervención II, totalizando 216 casos (Figura 1).

**Figura 1 -:** Flujograma de revisión de historias clínicas y estimación de prevalencia de eventos adversos antes y después de la implementación de listas de verificación quirúrgicas. Curitiba, PR, Brasil, 2022

*pEA = Potencial evento adverso; ^†^EA = Evento adverso

Hubo una reducción en el número de pacientes afectados por dos o más EA, con una variación del 27,8 % (pre-intervención) al 11,3 % (p = 0,002) en el post-intervención II. La Tabla 3 presenta los EA según su clasificación.

**Tabla 3 -:** Distribución de los eventos adversos antes y después de la implementación de listas de verificación quirúrgicas según clasificación, grado de daño y evitabilidad. Curitiba, PR, Brasil, 2022

**Variable**	**Período**						**Total (n= 216)**	
	Pre-intervención (n= 91)		Post-intervención I (n= 75)		Post-intervención II (n= 50)			
	n	%	n	%	n	%	n	%
**Clasificación del evento adverso**								
Medicación/fluidos intravenosos	41	45	21	28	7	14	69	31,9
Proceso/procedimiento clínico	32	35,2	36	48	29	58	97	44,9
Dispositivos/equipos médicos	8	8,8	11	14,7	6	12	25	11,6
Infección relacionada con la asistencia sanitaria	7	7,7	4	5,4	5	10	16	7,4
Administración clínica	2	2,2	1	1,3	1	2	4	1,9
Accidentes con el paciente	1	1,1	1	1,3	0	0	2	0,9
Sangre/hemoderivados	0	0	0	0	2	4	2	0,9
Infraestructura/lugar/instalaciones	0	0	1	1,3	0	0	1	0,5
**Grado de daño**								
Leve	62	68,1	60	80	35	70	157	72,7
Moderado	24	26,4	15	20	10	20	49	22,7
Grave	4	4,4	0	0	5	10	9	4,2
Óbito	1	1,1	0	0	0	0	1	0,4
**Potencial de evitabilidad**								
Fuertemente evitable	10	11	20	26,7	15	30	45	20,8
Potencialmente evitable	72	79,1	47	62,7	29	58	148	68,5
Potencialmente no evitable	9	9,9	8	10,7	4	8	21	9,7
Fuertemente no evitable	0	0	0	0	2	4	2	0,1

La clasificación de los tipos de EA ocurridos antes y después de las intervenciones se presenta en la Tabla 4. Se observó, entre los períodos pre-intervención y post-intervención I y II, una reducción significativa en la prevalencia de pacientes afectados por retención urinaria (33%; 14,4%; 3,1%, p < 0,001) y por hemorragia (9,3%; 7,2%; 0%, p = 0,012).

Lo contrario ocurrió con los casos de lesiones cutáneas (lesión por posicionamiento quirúrgico/lesión por dispositivo/adhesivo médico), que aumentaron del 2,1% (pre-intervención) al 10,3% (post-intervención) (p = 0,043). No se identificó una diferencia significativa en la prevalencia de pacientes con infección de herida quirúrgica (6,2%; 4,1%; 5,2%, p = 0,810) ni hematoma/seroma (5,2%; 9,3%; 8,3%, p = 0,528).

**Tabla 4 -:** Distribución de los eventos adversos antes y después de la implementación de listas de verificación quirúrgicas según el tipo. Curitiba, PR, Brasil, 2022

**Tipo de evento adverso**	**Periodo de intervención**						**Total (n= 216)**	
	Pre-intervención (n= 91)		Post-intervención I (n= 75)		Post-intervención II (n= 50)			
	N	%	N	%	N	%	n	%
Retención urinaria	32	35,2	14	%	3	6	49	22,7
Hematoma/seroma	5	5,5	9	12	8	16	22	10,2
Hemorragia	9	9,9	7	9,3	0	0	16	7,4
Desplazamiento de catéter epidural	7	7,7	7	9,3	1	2	15	6,9
Infección de la herida	6	6,6	4	5,3	5	10	15	6,9
Lesión de la mucosa/piel	2	2,2	7	9,3	6	12	15	6,9
Náuseas/vómitos	6	6,6	3	4	2	4	11	5,1
Lesión por posicionamiento quirúrgico	0	0	4	5,3	5	10	9	4,2
Trombosis venosa profunda	3	3,3	2	2,7	2	4	7	3,2
Falta de material/equipo/cama	2	2,2	2	2,7	1	2	5	2,3
Dolor agudo	0	0	4	5,3	0	0	4	1,9
Fractura	3	3,3	1	1,3	0	0	4	1,9
Luxación	1	1,1	0	0	3	6	4	1,9
Neuropraxia	1	1,1	3	4	0	0	4	1,9
Prurito	0	0	3	4	1	2	4	1,9
Dehiscencia de sutura	1	1,1	1	1,3	1	2	3	1,4
Dismetría de miembro inferior	2	2,2	1	1,3	0	0	3	1,4
Fallo del bloqueo anestésico	1	1,1	0	0	2	4	3	1,4
Fallo del equipo	1	1,1	0	0	2	4	3	1,4
Aflojamiento del material de síntesis	1	1,1	1	1,3	1	2	3	1,4
Tromboembolia pulmonar	0	0	0	0	3	6	3	1,4
Hipotensión	2	2,2	0	0	0	0	2	0,9
Perforación de la duramadre	1	1,1	1	1,3	0	0	2	0,9
Reacción transfusional	0	0	0	0	2	2	2	0,9
Accidente cerebrovascular	1	1,1	0	0	0	0	1	0,5
Choque séptico	1	1,1	0	0	0	0	1	0,5
Enfisema subcutáneo	1	1,1	0	0	0	0	1	0,5
Error de medicación	1	1,1	0	0	0	0	1	0,5
Fístula	0	0	0	0	1	2	1	0,5
Hipoglucemia	1	1,1	0	0	0	0	1	0,5
Hipoxia	0	0	0	0	1	2	1	0,5
Intoxicación por opioide	0	0	1	1,3	0	0	1	0,5

En cuanto a la frecuencia de readmisiones debido a EA, no se observó una diferencia significativa antes y después de la implementación de las listas de verificación (p = 0,092): pre-intervención (9,7%; n = 6), post-intervención I (8,2%; n = 4) y post-intervención II (22,9%; n = 8).

## Discusión

La reducción de la prevalencia de EA entre pacientes sometidos a artroplastia de cadera y rodilla requiere comprender los distintos factores que contribuyen al aumento de los riesgos asistenciales y quirúrgicos, así como un juicio crítico por parte de los gestores respecto a las intervenciones previstas en la planificación estratégica institucional para promover prácticas seguras y proteger a los pacientes de errores y daños quirúrgicos. En esta investigación se constató un descenso significativo en la prevalencia y el número de eventos adversos por paciente tras la aplicación de las dos listas de verificación quirúrgicas. Estos resultados se suman a la evidencia que indica que el uso de listas de verificación de seguridad quirúrgica, o *checklists*, ha favorecido prácticas seguras y basadas en valor^(28)^.

El resultado favorable presentado en esta investigación es potencialmente fruto del esfuerzo de los profesionales de la salud por adaptar, elaborar e implementar gradualmente listas de verificación quirúrgicas para cumplir con las recomendaciones del PCSSV, así como de la participación de los líderes en la construcción progresiva de una cultura de seguridad del paciente, un factor que contribuye a la visibilidad de los riesgos y a la adopción de acciones preventivas.

Resultados satisfactorios a partir de la implementación de la lista de verificación quirúrgica también fueron observados en un estudio realizado en un hospital terciario de Australia, cuyos autores señalaron una reducción significativa en la tasa de mortalidad del 1,2% al 0,92%^(29)^. De manera similar, una investigación llevada a cabo en hospitales universitarios del Reino Unido mostró una disminución en la prevalencia de EA, del 16,9% al 11,2% (p = 0,01)^(30)^.

En Noruega, investigadores señalaron que el uso concomitante de una lista de verificación aplicada en el pre y postoperatorio, junto con la lista de verificación utilizada en el intraoperatorio, redujo significativamente los EA, las reoperaciones y las readmisiones no planificadas^(31)^. Este dato difiere, en parte, de los resultados de la presente investigación, en la que no se observaron diferencias en las readmisiones derivadas de estos eventos. Cabe destacar que los efectos positivos de las listas de verificación también se evidencian en servicios quirúrgicos de países de ingresos bajos y medianos, como es el caso de Brasil, con una reducción del 44% en la incidencia general de EA^(32)^, lo que respalda los hallazgos aquí presentados.

En esta investigación, la reducción significativa de pacientes con retención urinaria tras las intervenciones puede explicarse principalmente por la disminución observada en el uso de anestesia epidural y analgesia aguda mediante catéter epidural en la fase postoperatoria. Otro factor potencialmente relacionado es la adhesión a las directrices basadas en evidencia para prevenir este evento, mediante el uso cauteloso de opioides administrados por vía intratecal y la combinación de distintas drogas analgésicas para el tratamiento del dolor agudo^(33-34)^, incluso desde el período preoperatorio, con el objetivo de reducir el consumo de opioides en el postoperatorio^(35)^.

Se reitera que la adopción de la lista de verificación permite mejorar la planificación preoperatoria y anticipar eventos críticos mediante la identificación de factores intrínsecos y extrínsecos que incrementan el riesgo clínico-quirúrgico de episodios de retención urinaria^(36)^. Este hallazgo es relevante, ya que dicho EA potencializa problemas relacionados con el músculo vesical, comprometiendo su funcionalidad y aumentando el riesgo de infecciones urinarias, lo que conlleva la necesidad de procedimientos complementarios y el consecuente incremento de los riesgos y del tiempo de hospitalización^(36-37)^.

En cuanto a los impactos de la lista de verificación en la prevención de hemorragias, se observan divergencias en la literatura al considerar los distintos escenarios perioperatorios. Mientras un estudio realizado en Noruega reveló una disminución de los casos de sangrado posoperatorio del 2,6% al 1,0% (p< 0,001), así como de los costos asociados a la transfusión de hemocomponentes^(38)^, otra investigación llevada a cabo en un centro de cuidados terciarios en Australia constató que no hubo diferencia en la ocurrencia de este EA^(29)^.

La diferencia significativa encontrada en esta investigación respecto al número de pacientes expuestos a este EA, antes y después de las intervenciones, puede estar relacionada con mejoras en la evaluación del paciente, el uso de equipos y las técnicas operatorias empleadas. Se reconoce que el PCSSV, en la etapa preoperatoria inmediata, busca anticipar la posibilidad de pérdida sanguínea y permitir la planificación de acciones multimodales, como la reserva previa de factores lábiles de coagulación y hemocomponentes, con el objetivo de minimizar y tratar esta eventualidad, además de evitar EA graves^(39)^. Así, considerando que se trata de cirugías electivas, la evaluación realizada por los equipos quirúrgico, de anestesiología y de enfermería en el preoperatorio ofrece una oportunidad para la prevención del daño.

También debe considerarse que se trata de un hospital de enseñanza y, como tal, la escasa experiencia de los residentes en la realización de técnicas operatorias representa un factor de riesgo para el paciente y un desafío para los coordinadores y profesionales que integran los núcleos de seguridad del paciente. La supervisión directa de los aprendices contribuye a una formación de calidad y a un aprendizaje seguro, promoviendo la seguridad del paciente mediante la optimización del tiempo operatorio en las artroplastias de cadera y rodilla. Además, mejoras relacionadas con la gestión del cuidado de enfermería en el posoperatorio, como la evaluación y manejo de apósitos y drenajes quirúrgicos, así como la aplicación de crioterapia, pueden haber contribuido a la reducción de los eventos hemorrágicos^(40)^. La lista de verificación institucional, aplicada en el período posoperatorio inmediato, incluye la verificación de signos de sangrado, cuya detección precoz contribuye a evitar el agravamiento del caso^(19)^.

La implementación de las dos modalidades de lista de verificación no influyó en la reducción del tiempo de internación hospitalaria, contrariamente a lo señalado en la literatura^(29)^. Se infiere que la mayor gravedad y complejidad de los pacientes pueden haber contribuido al mantenimiento del tiempo del proceso anestésico-quirúrgico y de la estancia hospitalaria. Asimismo, se constató un aumento en la prevalencia de pacientes con daños en el tejido cutáneo, caracterizados como lesiones por posicionamiento quirúrgico y/o lesiones causadas por dispositivos o adhesivos médicos. Diversos factores pueden haber contribuido a la ocurrencia de estos eventos en la muestra estudiada, los cuales podrían ser explorados en investigaciones posteriores. No obstante, la reducción del tiempo operatorio^(41)^ y el uso de dispositivos y superficies de soporte adecuados^(42-43)^ son factores protectores contra estos EA que merecen ser abordados desde la gestión, a la luz de la formación y práctica profesional, con especial atención al equipo de enfermería.

Por otro lado, cabe destacar que, antes de las intervenciones, estos agravantes podrían haber sido percibidos por el equipo de salud como ocurrencias comunes en el ámbito quirúrgico, recibiendo, en consecuencia, menor importancia en cuanto al registro y notificación en comparación con otros EA. Con el auge de los aspectos relacionados con la gestión de riesgos y la mejora de los protocolos para la prevención de incidentes quirúrgicos, probablemente se fomentó el desarrollo de una cultura de seguridad del paciente a lo largo del período de la investigación. Este avance puede haber contribuido a mejoras en los procesos de trabajo, aumentando la percepción y el compromiso de los profesionales con la identificación y el registro de estos EA. Como consecuencia, se hizo posible detectar, en la revisión documental, eventos previamente no registrados en las historias clínicas de los pacientes.

No se identificó una disminución significativa en la prevalencia de pacientes con infección de herida quirúrgica y hematomas/seromas, a diferencia de lo constatado en hospitales de Etiopía, donde la prevalencia de ISQ pasó del 27,3% al 9,1% (p= 0,086)^(44)^, y de lo informado por investigadores de la India, quienes observaron una reducción significativa de este tipo de evento adverso antes y después de la implementación de la lista de verificación (del 14% al 9,5%)^(45)^. Sin embargo, se reconoce que el uso de estas herramientas de forma aislada tiene un efecto limitado como medida de prevención de ISQ^(46)^, ya que su ocurrencia es de naturaleza multifactorial, involucrando factores relacionados con el entorno, el paciente y el procedimiento quirúrgico^(47)^.

No obstante, se observa que la prevalencia de EA sigue siendo elevada en el hospital del estudio, siendo la mayoría de estos eventos evitables, conforme un estudio de cohorte multicéntrico realizado en 24 hospitales de Suecia, que reportó una prevalencia de EA en artroplastias de cadera del 58,7%, con un 76% de los registros clasificados como prevenibles^(48)^. En Brasil, los estudios epidemiológicos para evaluar la gravedad y evitabilidad de estos eventos, exclusivamente en el área ortopédica, son incipientes. Sin embargo, se observa que una incidencia de EA del 33,7% fue evidenciada en un hospital de enseñanza situado en la región sureste de Brasil, con más de la mitad de los casos siendo evitables^(49)^.

Los resultados presentados en esta investigación contribuyen a evidenciar el potencial de la implementación de listas de verificación en la reducción de EA y reiteran la importancia de adoptar buenas prácticas asistenciales y su monitoreo a través de estudios epidemiológicos. Otro aspecto contribuyente de esta investigación al conocimiento científico está relacionado con la metodología de rastreo de eventos adversos adoptada, que es replicable en diferentes culturas y contextos asistenciales. La identificación activa de EA contribuye a reconocer y clasificar desenlaces desfavorables, a la construcción de indicadores de calidad y a respaldar la planificación y revisión de protocolos asistenciales.

Asimismo, los resultados contribuyen a incentivar a otros investigadores a explorar el tema en distintos contextos, dado que el uso de estas herramientas está fuertemente influenciado por las particularidades de cada unidad asistencial, considerando aspectos tecnológicos, demográficos, epidemiológicos y los modelos de gestión de las organizaciones de salud, además de la adhesión del equipo multidisciplinario a los principios de calidad.

Entre las limitaciones de la presente investigación se destacan el análisis retrospectivo basado en documentos, que depende de la calidad de los registros, el tamaño de la muestra y la realización del estudio en un único hospital. Igualmente, se reconoce que múltiples factores pueden influir en la calidad asistencial y en la ocurrencia de EA, sin limitarse a las intervenciones aquí estudiadas.

## Conclusión

Hubo una reducción en la frecuencia y prevalencia general de eventos adversos tras la implementación de listas de verificación quirúrgicas, especialmente en la disminución de la retención urinaria y las hemorragias. Sin embargo, se constató un aumento en la prevalencia de lesiones cutáneas. No se observaron efectos relacionados con la ocurrencia de infecciones de herida quirúrgica ni de hematomas/seromas, así como tampoco en el tiempo de hospitalización ni en las readmisiones hospitalarias derivadas de eventos adversos.
